# Didymin alleviates metabolic dysfunction-associated fatty liver disease (MAFLD) via the stimulation of Sirt1-mediated lipophagy and mitochondrial biogenesis

**DOI:** 10.1186/s12967-023-04790-4

**Published:** 2023-12-19

**Authors:** Jing-wen Yang, Ying Zou, Jun Chen, Chen Cui, Jia Song, Meng-meng Yang, Jing Gao, Hui-qing Hu, Long-qing Xia, Li-ming Wang, Xiao-yu Lv, Li Chen, Xin-guo Hou

**Affiliations:** 1 Department of Endocrinology, Qilu Hospital of Shandong University, Cheeloo College of Medicine, Shandong University, Jinan, 250012 Shandong China; 2 Department of Endocrinology, The Second Hospital of Shandong University, Jinan, China; 3 Key Laboratory of Endocrine and Metabolic Diseases, Shandong Province Medicine & Health, Jinan, China; 4 Jinan Clinical Research Center for Endocrine and Metabolic Disease, Jinan, China; 5 Institute of Endocrine and Metabolic Diseases, Shandong University, Jinan, China; 6 National Key Laboratory for Innovation and Transformation of Luobing Theory, Jinan, China; 7 The Key Laboratory of Cardiovascular Remodeling and Function Research, Chinese Ministry of Education, Chinese National Health Commission and Chinese Academy of Medical Sciences, Jinan, China

**Keywords:** Didymin, Sirt1, MAFLD, Mitochondrial function, Lipophagy, Apoptosis, PGC-1α, FoxO3a

## Abstract

**Background:**

Metabolic dysfunction-associated fatty liver disease (MAFLD) is one of the most prevalent metabolic syndromes worldwide. However, no approved pharmacological treatments are available for MAFLD. Chenpi, one kind of dried peel of citrus fruits, has traditionally been utilized as a medicinal herb for liver diseases. Didymin is a newly identified oral bioactive dietary flavonoid glycoside derived from Chenpi. In this study, we investigated the therapeutic potential of Didymin as an anti-MAFLD drug and elucidated its underlying mechanisms.

**Methods:**

High-fat diet (HFD)-induced MAFLD mice and alpha mouse liver 12 (AML12) cells were utilized to evaluate the effects and mechanisms of Didymin in the treatment of MAFLD. Liver weight, serum biochemical parameters, and liver morphology were examined to demonstrate the therapeutic efficacy of Didymin in MAFLD treatment. RNA-seq analysis was performed to identify potential pathways that could be affected by Didymin. The impact of Didymin on Sirt1 was corroborated through western blot, molecular docking analysis, microscale thermophoresis (MST), and deacetylase activity assay. Then, a Sirt1 inhibitor (EX-527) was utilized to confirm that Didymin alleviates MAFLD via Sirt1. Western blot and additional assays were used to investigate the underlying mechanisms.

**Results:**

Our results suggested that Didymin may possess therapeutic potential against MAFLD in vitro and in vivo. By promoting Sirt1 expression as well as directly binding to and activating Sirt1, Didymin triggers downstream pathways that enhance mitochondrial biogenesis and function while reducing apoptosis and enhancing lipophagy.

**Conclusions:**

These suggest that Didymin could be a promising medication for MAFLD treatment. Furthermore, its therapeutic effects are mediated by Sirt1.

**Supplementary Information:**

The online version contains supplementary material available at 10.1186/s12967-023-04790-4.

## Introduction

MAFLD is a leading cause of chronic liver disease, with a global prevalence of 24%, and is still increasing due to the rising prevalence of obesity globally [1]. Despite several research studies supporting its treatment, the United States Food and Drug Administration (US-FDA) and the European Medicines Agency haven't approved any specific medications for NAFLD [2–6]. Some traditional Chinese medicines have demonstrated benefits in treating liver diseases [7–10]. Exploring the main components that perform a therapeutic role is a promising step toward developing a viable drug for MAFLD treatment.

**Fig. 1:**
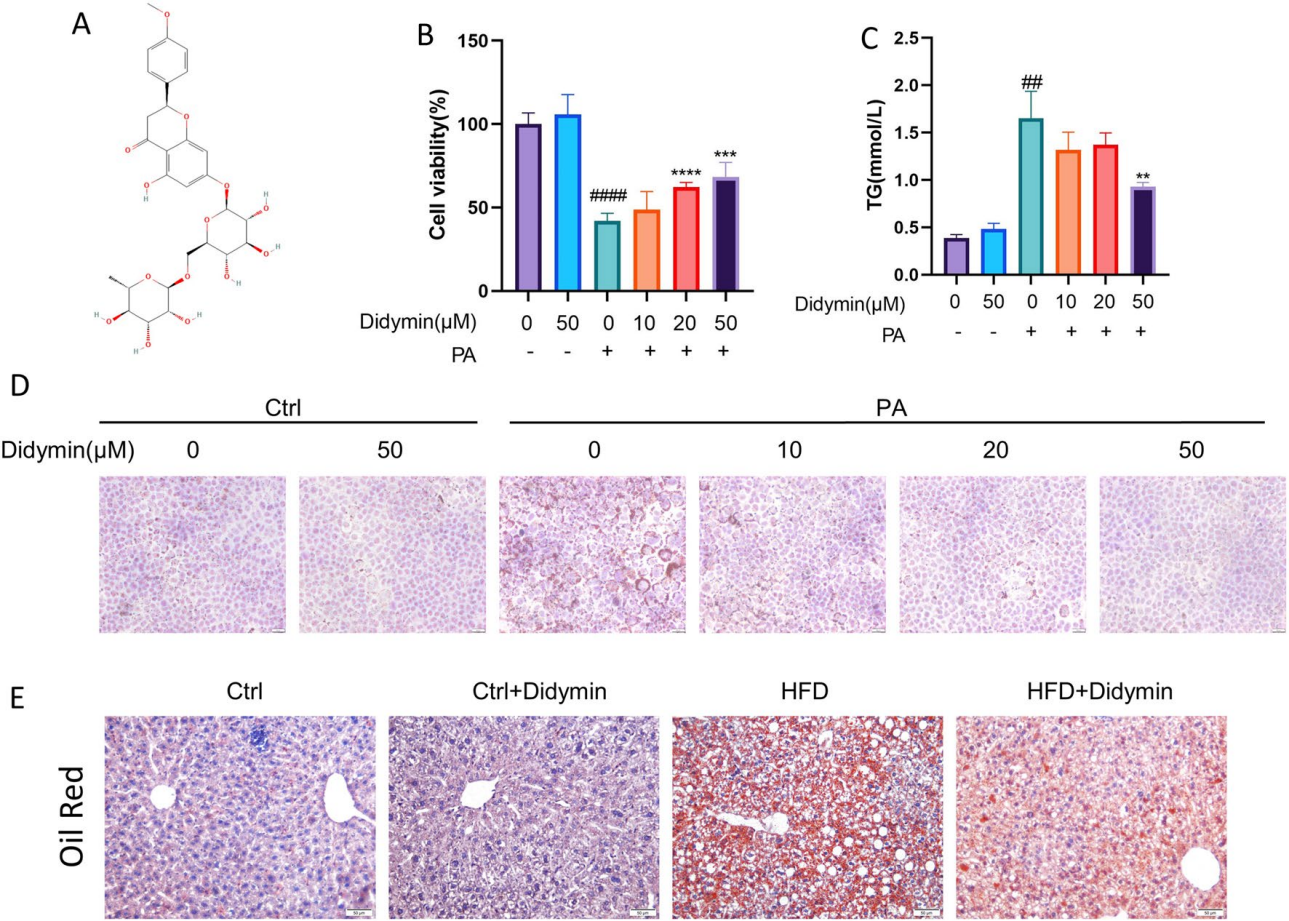
Didymin suppresses high-fat-induced hepatocellular lipid deposition both in vitro and in vivo*.* **A** Chemical structure of Didymin. **B** Cell viability of AML12 cells treated with PA and different concentrations of Didymin (n = 4). **C** TG contents in AML12 cells (n = 4). **D** Lipid accumulation in AML12 cells was visualized using oil red O staining (Scale bar = 20 μm). **E** A group of MAFLD mice fed with a high-fat diet for 20 weeks and a control group fed with a normal diet were randomly divided into two groups each. After three weeks of Didymin treatment, the lipid deposition in the livers of the four groups of mice was visualized using oil red O staining. Data are expressed as mean ± SD. **P < 0.01, ***P < 0.001, ****P < 0.0001 PA vs. PA + Didymin. ^##^P < 0.01, ^####^P < 0.0001 control vs. PA. *PA* palmitic acid, *TG* triglyceride

The dried peel of *citrus* fruits (known as *Citri Reticulatae Pericarpium*, Chenpi) has traditionally been used in Chinese medicine as a medicinal herb for liver diseases [11]. Chenpi, rich in flavonoids, is known for its medicinal properties [12]. Didymin (isosakuranetin 7-O-rutinoside) **(**Fig. 1A**)** is an orally bioactive flavonoid glycoside found in various citrus fruits [13–17]. Due to its high citrus content and ease of extraction, Didymin has been recognized as a cost-efficient and safe oral treatment [18]. Recent studies have demonstrated promising biological activities of Didymin, including anticancer [14, 18, 19], antioxidant and neuroprotective [20], hepatoprotective [20], antinociceptive [21], anti-inflammation [22], and cardiovascular effects [23]. Although its anti-cancer and anti-oxidative characteristics have been proven in numerous diseases, the potential of Didymin in anti-lipotoxicity has not yet been studied.

In this study, we investigated the hepatoprotective effects of Didymin against MAFLD in both in vitro AML12 cells and in vivo HFD-induced MAFLD mice. We further elucidated the underlying mechanisms and demonstrated that Didymin ameliorated mitochondrial function, augmented lipophagy, and attenuated apoptosis by enhancing the expression and deacylation activity of Sirt1.

## Materials and methods

### Cell culture and transfection

The AML12 cells were purchased from the American Type Culture Collection (Rockville, MD, USA). Cells were cultured in DMEM/F12 medium, supplemented with 10% FBS, 1% Penicillin–Streptomycin solution, 40 ng/mL dexamethasone, and ITS (10 µg/mL insulin, 5.5 µg/mL transferrin, 6.7 μg/L Sodium Selenite, Beyotime, C0314). The cells were cultured in a humidified environment with 5% CO_2_ at a temperature of 37 °C. Palmitic acid (PA) is the most abundant saturated fatty acid in liver triglycerides [24]. It is more likely to induce lipotoxicity of MAFLD [25, 26], which is widely used in the simulation of lipotoxicity in vitro models [27–31]. AML12 cells underwent different treatments as described below: (1) Control groups: without Didymin or PA stimulated; (2) Control + Didymin groups: cells were incubated with Didymin (50 μM, MedChemExpress, HY-N2068), without PA for a duration of 24 h. Based on previous research [32, 33] and the results of our pre-experiments, we selected Didymin stimulus for 50 μM for 24 h. (3) PA groups: The cells were subjected to a 24 h treatment by PA (0.5 mM) lacking Didymin. Based on previous research [27–31] and the results of our pre-experiments, we selected PA stimulus for 0.5 mM for 24 h. (4) PA + Didymin groups: cells underwent treatment with PA (0.5 mM) and Didymin (50 μM) simultaneously for 24 h. (5) PA + EX-527 groups: cells underwent treatment with PA (0.5 mM) and EX-527 (10 μM, MedChemExpress, New Jersey, USA, HY-15452) simultaneously for 24 h. (6) PA + EX-527 + Didymin groups: cells underwent treatment with PA at a concentration of (0.5 mM), EX-527 (10 μM), and Didymin (50 μM) simultaneously for 24 h. The cells were transiently transfected employing Lipofectamine 3000 reagent (Invitrogen, Grand Island, USA, L3000075) based on the manufacturer's guidelines.

### Cell viability

The cells were inoculated into a 96-well plate and subjected to the aforementioned treatment. The determination of viable cells was performed utilizing the Cell Counting Assay-8 kit (CCK-8; Biosharp, Beijing, China, BS350A). Once the cells had adhered adequately, 20 μL of CCK-8 was introduced into each well and allowed to incubate for a duration of 1 h. Subsequently, the outcomes were documented in terms of absorbance optical density at a wavelength of 450 nm.

### RNA-seq analysis

Total RNA isolation was carried out using Trizol reagent (Invitrogen, Grand Island, USA, 15596026) with respect to the manufacturer's protocol. The library construction and sequencing procedures were conducted at Shenzhen BGI Genomics Co. Data mining analysis was conducted utilizing Dr. Tom multiple omics data mining system (https://biosys.bgi.com).

### Immunoprecipitation

Protein A/G magnetic beads (MedChemExpress, New Jersey, USA, HY-K0202) were washed three times with RIPA lysis buffer (Beyotime, Shanghai, China, P0013B) three times before IP. The primary antibody was subjected to incubation with protein A/G magnetic beads for a duration of 1 h at a temperature of 4 °C while being gently mixed. Cells were subjected to two rounds of cold PBS washing (Beyotime, Shanghai, China, C0221A) and were subjected to lysis using RIPA lysis buffer supplemented with a cocktail of deacetylase inhibitors (Beyotime, Shanghai, China, P1112) and phenylmethanesulfonyl fluoride (PMSF, Beyotime, Shanghai, China, ST506) for 30 min at 4 °C. The cellular lysates underwent centrifugation at 13,000*g* for 10 min, following which the resulting supernatants were carefully transferred into fresh tubes. Subsequently, the cellular lysate was subjected to an incubation process with a mixture of antibody beads while being maintained at a temperature of 4 °C and subjected to rotary agitation overnight. The immune complex underwent a triple wash with RIPA lysis buffer and was subsequently subjected to boiling in protein loading buffer (Beyotime, Shanghai, China, P0015) for 5 min at a temperature of 95 °C. Subsequently, the immunoprecipitate was analyzed by Western blotting.

### Western blot analysis

Cells were rinsed with two rounds of cold PBS, followed by lysis in RIPA lysis buffer introduced with a deacetylase inhibitor cocktail and PMSF for 30 min at a temperature of 4 °C. Cell lysates were centrifuged at 13,000*g* for 10 min, after which the resulting supernatants were carefully transferred into fresh tubes. The quantification of protein concentration for each sample was performed utilizing a BCA protein assay kit (Beyotime, Shanghai, China, P0010). An equivalent quantity of protein was separated using 12.5% SDS-PAGE (Epizyme Biomedical Technology, Shanghai, China, PG113), transferred to PVDF membranes (Millipore, Massachusetts, USA, ISEQ00010), blocked with 5% nonfat milk, and underwent incubation with specific primary antibody at 4 °C overnight. Following a triple wash with TBST (100 mM NaCl, 10 mM Tris–HCl, pH 7.5, and 0.1% Tween‐20), membranes were incubated with secondary antibody conjugated with horseradish peroxidase at room temperature for a duration of 2 h. The proteins were observed through the utilization of enhanced chemiluminescence (Millipore, Massachusetts, USA, WBKLS0500). Table 1 presents all primary antibodies used in the western blot.

**Table 1:** Antibodies

Antibody	Source	Vendor	Catalog no.	Dilution factor
Sirt1	Rabbit	Proteintech	13161-1-AP	1:1000
Pgc-1α	Mouse	Proteintech	66369-1-Ig	1:1000
FoxO3a	Rabbit	Cell Signaling Technology	#2497	1:1000
p-FoxO3a (Ser253)	Rabbit	Cell Signaling Technology	#9466	1:500
Acetylated-Lysine	Rabbit	Cell Signaling Technology	#9441	1:500
Bax	Rabbit	Cell Signaling Technology	#2772	1:1000
Bcl-2	Mouse	ImmunoWay	YM3041	1:1000
caspase-3	Rabbit	Cell Signaling Technology	#9662	1:1000
cleaved caspase-3 (Asp175)	Rabbit	Cell Signaling Technology	#9661	1:1000
PARP	Rabbit	Cell Signaling Technology	#9532	1:1000
Cleaved-PARP	Rabbit	Cell Signaling Technology	#94885	1:500
LC3A/B	Rabbit	Cell Signaling Technology	#4108	1:1000
Beclin1	Rabbit	Cell Signaling Technology	#3495	1:1000
p62	Rabbit	ABcam	ab91526	1:1000
ATG5	Rabbit	Proteintech	10181-2-ap	1:1000
PLIN2	Rabbit	Proteintech	15294-1-AP	1:1000
LAMP1	Mouse	Cell Signaling Technology	15665	1:1000
NRF1	Rabbit	Proteintech	12482-1-AP	1:1000
TFAM	Rabbit	Proteintech	22586-1-AP	1:1000
NDUFB8	Rabbit	Proteintech	14794-1-AP	1:1000
MT-CO2	Rabbit	Cell Signaling Technology	#31219	1:1000
SDHB	Rabbit	Cell Signaling Technology	#92649	1:1000
HSP90	Rabbit	Proteintech	13171-1-AP	1:5000
β-Tubulin	Rabbit	Proteintech	10068-1-AP	1:10,000

### Molecular docking study

The crystallographic structure of Sirt1 utilized in this investigation was acquired from Brookhaven Protein Data Bank. The PDB entry is 4ZZH [34]. The structure of Didymin was obtained from the ChemSpider database (ChemSpider ID: 16498764) and underwent treatment as a ligand. The protein structure was methodically prepared through the addition of hydrogen, optimization of H-bond assignment, bond order assignment, disulfide treatment, and energy minimization to achieve structural relaxation. All bonds of ligands were set as rotatable. The Discovery Studio 2019 software was utilized to conduct the docking study. The ligand found in the crystal structure was utilized to identify the central location of a docking grid box, and the XYZ dimensions of the docking grid box were 25.350488, − 25.762176, − 25.387112.

### MST

The purified recombinant Sirt1 protein (MedChemExpress, New Jersey, USA, HY-P71596) was dissolved in PBS. Subsequently, they were labeled based on the Protein labeling kit RED-tris-NTA protocol (Nanotemper, Munich, Germany, L018). Didymin (100 μM) dissolved in DMSO was also diluted in PBS for the ultimate MST assay. The Monolith NT.115 instrument was utilized to conduct the MST experiment (NanoTemper Technologies, Munich, Germany). Proteins labeled at a concentration of 50 nM were combined with candidate compounds at the specified concentrations in a reaction buffer consisting of 20 mM HEPES at a pH of 7.4 and 150 mM NaCl. Subsequently, the MST data were acquired utilizing 100% infrared laser power and medium light-emitting diode power. The Nanotemper analysis software was utilized for the analysis of data (v.1.5.41), and the Kd was identified.

### Sirt1 deacetylase activity

Quantification of Sirt1 deacetylase activity was performed using the fluorometric Sirt1 assay kit (Abcam, Cambridge, UK, ab156065) based on the manufacturer's guidelines. The fluorescence intensity was monitored using an excitation wavelength of 355 nm and an emission wavelength of 450 nm every 2 min. In testing overall Sirt1 protein deacetylase activity in cells, data were subsequently normalized by the amount of protein. The fold representation of overall Sirt1 deacetylase activity in cells treated with Didymin was contrasted with that of the vehicle control group. The fold representation of recombinant Sirt1 deacetylase activity treated with Didymin was contrasted with that of the control (PMSF) group.

### MitoTracker green staining

The cells were subjected to mitochondrial labeling using MitoTracker Green probes (Beyotime, Shanghai, China, C1048) based on the manufacturer's guidelines. Afterward, the live cells that had been labeled were analyzed using confocal imaging to quantify the mitochondria present. Fluorescence intensity was analyzed by Image J software and corrected by protein concentration.

### Seahorse analysis

A Seahorse Bioscience XF24-3 extracellular flux analyzer (Agilent, Santa Clara, CA) was utilized for the measurement of oxygen consumption rate (OCR). The evaluation of OCR was conducted using an XF base medium containing glucose (10 mM), pyruvate glutamine (2 mM), and pyruvate (1 mM) in accordance with the instructions provided by the manufacturer. The experimental procedures involved the utilization of 1.5 μM oligomycin, 2 μM carbonyl cyanide-4-(trifluoromethoxy) phenylhydrazone (FCCP), and 0.5 μM antimycin A/rotenone for the analyses. The normalization of data was performed based on the quantity of protein. The outcomes were examined utilizing the WAVE software and subjected to the XF Mito Stress Test Report for processing.

### Plasmid construction

The structural integrity of green fluorescent protein (GFP) is compromised in the lumen of the lysosome under acidic and proteolytic conditions, whereas mCherry exhibits greater stability and is detectable within the lysosome. Hence, the colocalization of GFP and mCherry fluorescence is deemed to signify a compartment that has not undergone fusion with a lysosome; mCherry only represents lipophagy [35]. Tandem mCherry-GFP fluorescence microscopy is useful in the monitoring of degradation processes associated with lysosomes [36], and the protein perilipin 2 (PLIN2) has been identified to be located on the surface of lipid droplets (LDs) in hepatocytes [37]. Through the combination of tandem mCherry-GFP microscopy and PLIN2, a tandem mCherry-GFP-PLIN2 fusion protein was created to facilitate the observation of lysosomal degradation of LDs. The fluorescence number was analyzed by Image J software and corrected by cell count.

### Flow cytometry

The annexin V-FITC apoptosis detection kit (Yeasen Biotechnology, Shanghai, China, 40302ES60) was employed in the detection of cell apoptosis via flow cytometry in accordance with the guidelines. Adherent cells underwent digestion via trypsin without ethylenediaminetetraacetic acid (EDTA). Cells were washed with two rounds of cold PBS and resuspended in 400 μL of 1X binding solution at a concentration of around 1 × 106 cells/mL. Add 5 μL Annexin V-FITC staining solution incubate for 15 min, and then add 5 μL PI staining solution and incubate for 5 min in 4 °C dark conditions. The Beckman Coulter Gallios flow cytometer was utilized to promptly identify and evaluate both early and late apoptosis of cells.

### TUNEL

The measurement of 3′-OH terminus of fragmented DNA in apoptotic cells can be accomplished through TUNEL staining. The AML12 cells were subjected to the notch-end labeling approach, wherein they were fluorescently stained in accordance with the instructions provided by the TMR (red) Tunel Cell Apoptosis Detection Kit (Servicebio, Wuhan, China, G1502-100T). The cells underwent fixation by exposure to a 4% paraformaldehyde solution for 30 min. Subsequently, incubate cells with 0.3% Triton X-100 for 30 min for permeabilization. Then, incubate them with a TUNEL reaction solution at a temperature of 37 °C for 1 h. After incubation, stain them with DAPI for 10 min. Finally, the fluorescence microscope and Image J software were utilized to observe the images and determine the percentage of positively stained total cells.

### High-fat diet-induced MAFLD mice model

Four-weeks-old male C57BL/6J mice (Beijing SPF Biotechnology Co., Ltd., China), five mice in each cage, were kept in a standard SPF facility of Shandong University, where the temperature was maintained at 22 °C and a 12-h day and night cycle. After a one-week acclimation period, they were allocated into six groups (eight mice per group). All groups had access to food and drink. The experimental design is as follows: (1) Control group: had subjected to treatment with normal chow diet (NCD, Xietong Shengwu, Jiangsu, China, SWS9102) (12% fat, 20.6% protein, and 67.4% carbohydrate) for 23 weeks, and injected 10% DMSO + 90% corn oil without Didymin intraperitoneally in the last three weeks; (2) Control + Didymin group: treated with normal chow diet for 23 weeks, and injected Didymin 0.8 mg/kg (dissolve in 10% DMSO + 90% corn oil) intraperitoneally daily in the last three weeks; (3) High-fat diet (HFD) group: high-fat diet (Xietong Shengwu, Jiangsu, China, XTHF60) (60% fat, 20% carbohydrate, and 20% protein) for 23 weeks and injected 10% DMSO + 90% corn oil without Didymin intraperitoneally in the last three weeks; (4) HFD + Didymin group: treated with high-fat diet for a period of 23 weeks, and injected Didymin 0.8 mg/kg intraperitoneally daily in the last three weeks; (5) HFD + EX-527 group: high-fat diet for 23 weeks, and injected EX-527 5 mg/kg (dissolve in 10% DMSO + 90% corn oil) intraperitoneally every two days in the last three weeks; (6) HFD + Didymin + EX-527 group: treated with high-fat diet for a period of 23 weeks, and injected Didymin 0.8 mg/kg daily and EX-527 5 mg/kg every two days intraperitoneally in the last three weeks. Based on previous research [32, 33, 38–43] and the conversion of bioavailability between different administration methods [44], we chose the doses of Didymin and EX-527. Didymin and EX-527 were dissolved in DMSO first, and then DMSO was added to corn oil. The volume ratio of DMSO to corn oil was 1:9. Mix well to get a clear solution. For the last three weeks, weight was measured before the daily injection. One day after the final injection, the mice were sacrificed by decapitation under anesthesia. We collected blood samples and aliquoted sera, stored them at − 80 °C, dissected livers, weighed them, and took pictures of them. Each mouse liver tissue was divided into three parts. One portion was subjected to fixation using a 10% formalin solution to facilitate histological examination. Another portion was cryopreserved at a temperature of − 80 °C to enable protein extraction. A third portion was fixed using 4% paraformaldehyde to conduct transmission electron microscopy (TEM) analysis. Figure 6A depicts the experimental design. All animal experiments were approved by the Laboratory Animal Ethical and Welfare Committee of Shandong University Cheeloo College of Medicine (approval number: 23002).

### Serum assays

The levels of serum total cholesterol (TC), triglyceride (TG), glutamic-oxaloacetic transaminase (AST), glutamic-pyruvic transaminase (ALT), low-density lipoprotein (LDL), and high-density lipoprotein (HDL) were evaluated using the TG Triglyceride Kit (single agent GPO-PAP method, Jiancheng, Nanjing, China, A110-1-1), Total cholesterol assay kit (single agent GPO-PAP method, Jiancheng, Nanjing, China, A111-1-1), Alanine aminotransferase assay kit (Reitman Frankel assay, Jiancheng, Nanjing, China, C009-2-1), Aspartate aminotransferase assay kit (Jiancheng, Nanjing, China, C010-2-1), low-density lipoprotein cholesterol assay kit (Jiancheng, Nanjing, China, A113-1-1), and high-density lipoprotein cholesterol assay kit (Jiancheng, Nanjing, China, A112-1-1) respectively.

### Histological and transmission electron microscopy (TEM) analysis

Formaldehyde-fixed liver tissues were paraffin-embedded, sectioned into 5-μm-thick slices, and subsequently treated with hematoxylin and eosin (H&E) as well as periodic acid-Schiff (PAS) and Masson staining procedure was performed in accordance with established protocols. Standard protocols were employed for oil-red O staining using frozen sections.

Following the dewaxing of the slides, the process of antigen retrieval was carried out through the utilization of an antigen unmasking buffer for immunofluorescence staining. Following a 30 min blocking period at room temperature utilizing 10% normal goat serum, the slides were subjected to incubation with a primary antibody for γ-H2AX (ABcam, Cambridge, UK, ab81299, 1:100) at 4 °C overnight. Subsequently, the slides were subjected to incubation with a secondary fluorescent antibody (Zhongshan, Beijing, China, ZF-0311) at room temperature for 60 min and subsequently stained with DAPI for 5 min. Fluorescence microscopy was employed to visualize and capture fluorescence images (Olympus BX53, Japan).

The ultrastructure of hepatocytes was examined with TEM (JEM-1200EXT liver tissues were subjected to fixation using a 2.5% glutaraldehyde solution. Subsequently, the samples underwent two rounds of washing with 0.1 M phosphate buffer for 30 min each. Subsequently, they were fixed for a period of 2 h using a 1% solution of OSO_4_, subsequently by dehydration with 50%, 70%, 80%, and 90% ethanol. Samples underwent embedding with the epoxy resin mixture, and the blocks were sliced utilizing an ultramicrotome.

### Statistical analysis

The triplicate biological experiments data were expressed with error bars as mean ± SD. Two-tailed unpaired Student's t-test was utilized for the comparison of two groups of data. One-way ANOVA and Turkey test for adjustment was utilized for contrasting multiple data groups. A p-value of less than 0.05 was reported as significant.

## Results

### Didymin suppresses high-fat-induced hepatocellular lipid deposition both in vitro and in vivo

To investigate the role of Didymin in the pathogenesis of MAFLD, we established in vitro and in vivo models. The cytotoxicity of Didymin on AML12 cells was evaluated using the CCK8 assay, and it was found that even at a concentration of 50 μM, Didymin did not exhibit any apparent cytotoxic effects. However, Didymin dose-dependently prevented PA-induced cell death (Fig. 1B). Based on these findings, a concentration of 50 μM Didymin was selected for further in vitro studies. Moreover, Didymin treatment was found to inhibit lipid accumulation in AML12 cells cultured in PA-containing media, as demonstrated by reduced TG levels (about 43.81% reduced) (Fig. 1C). Consistent with these results, hepatic LDs were greatly increased by PA stimulation but dramatically decreased by Didymin treatment (Fig. 1D). Similarly, in the Oil Red O experiment, Didymin was shown to have a therapeutic impact on hepatocyte lipid accumulation in the livers of mice with MAFLD induced by HFD (Fig. 1E). Taken together, these findings indicate that Didymin suppresses high-fat-induced hepatocellular lipid deposition both in vitro and in vivo.

### Didymin protects AML12 cells against PA-induced lipid deposition by activating Sirt1

**Fig. 2:**
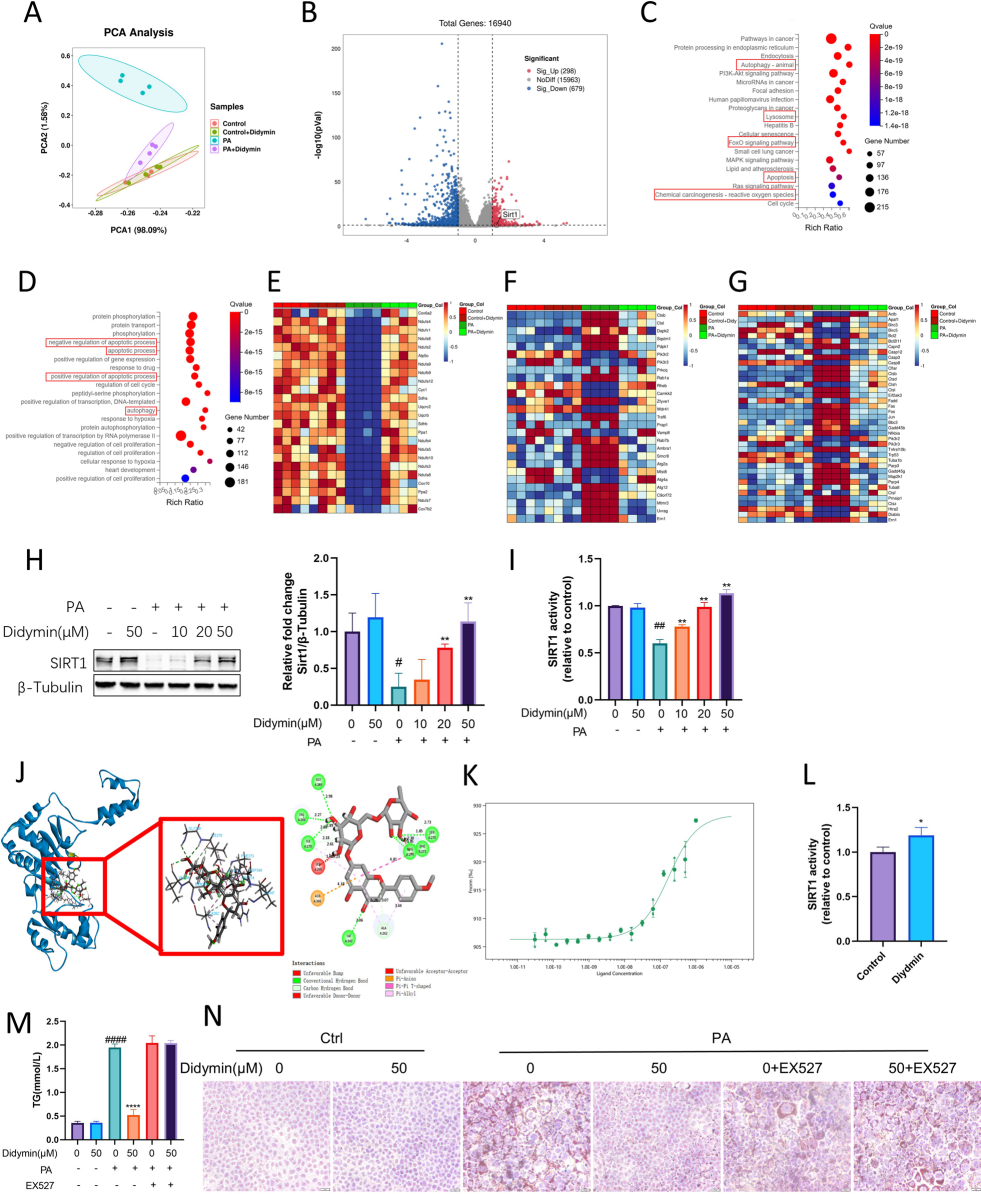
Didymin protects AML12 cells against PA‐induced lipid deposition by activating Sirt1. **A** Principal component analysis (PCA) of the RNA-sequencing data of AML12 cells. **B** Volcano-plot of RNA-seq results for PA + Didymin vs. PA. **C** KEGG analysis of the enrichment pathways. **D** GO Process (GO-P) analysis. Heatmaps of gene expression profiles related to (**E**) mitochondrial function, (**F**) autophagy, and (**G**) apoptosis based on the RNA-seq data set. (n = 4) (**H**) Western blot analysis of Sirt1 in AML12 cells (n = 3). **I** Sirt1 deacetylase activity in AML12 cells (n = 3). **J** Interactive sites between Didymin and Sirt1 by docking analysis. **K** MST analysis of the interaction between Didymin and Sirt1 (n = 3). **L** Sirt1 recombinant protein deacetylase activity (n = 4). **M** TG contents in AML12 cells (n = 4). **N** Oil red O staining of AML12 cells (Scale bar = 20 μm). Data are expressed as mean ± SD. *P < 0.05, **P < 0.01, ****P < 0.0001 PA vs. PA + Didymin. ^#^P < 0.05, ^##^P < 0.01, ^###^P < 0.001, ^####^P < 0.0001 control vs. PA. *PA* palmitic acid, *MST* MicroScale Thermophoresis, *TG* triglyceride

To elucidate the mechanism of action of Didymin on hepatocytes, we performed RNA sequencing analysis in AML12 cells. Unsupervised Principal Components Analysis (PCA) and hierarchical clustering of the PA-treated AML12 cells and the PA + Didymin co-treated AML12 cells clearly showed distinct clusters (Fig. 2A). We identified 977 differentially expressed genes (DEGs), with 298 genes upregulated in the PA + Didymin group compared to the PA group, and 679 genes downregulated (Fig. 2B). Pathway analysis using Kyoto Encyclopedia of Genes and Genomes (KEGG) revealed the abundance of genes related to autophagy, mitochondrial function, and apoptosis (Fig. 2C). GO Process (GO-P) analysis further confirmed the enrichment of genes involved in apoptotic and autophagy processes (Fig. 2D). Furthermore, heatmap analysis demonstrated significant alterations in the expression of genes related to mitochondrial function, autophagy, and apoptosis pathways upon PA treatment, which were mitigated by Didymin intervention (Fig. 2E–G).

Sirtuin 1 (Sirt1), an NAD^+^-dependent deacetylase, was reported as a crucial protein in the aforementioned biology processes such as mitochondrial function, autophagy, and apoptosis [45–47]. Furthermore, Sirt1 revealed higher expression in the PA + Didymin group compared to the PA group based on transcriptome sequencing data (Fig. 2B). Western blot results confirmed that PA treatment reduced Sirt1 levels in AML12 cells, while Didymin recovered Sirt1 levels in a dose-dependent manner (about 3.57 times elevated) (Fig. 2H). Therefore, we hypothesize that Sirt1 may be involved in the hepatoprotective process of Didymin. Next, we detected the overall Sirt1 deacetylase activity in the lysates of AML12 cells. The results showed that PA inhibited overall Sirt1 deacetylase activity, while Didymin dose-dependently increased Sirt1 deacetylase activity in PA-treated AML12 cells (about 88.98% elevated) Fig. 2I. We further investigated the mechanisms underlying the effect of Didymin on Sirt1 expression. Previous research has shown that Foxkhead Box Class O 3a (FoxO3a) is a transcription factor that regulates Sirt1 expression [48]. We knocked down the expression of FoxO3a using small interfering RNA in AML12 cells (Additional file 1: Fig. S1A). We found that the increase of Sirt1 expression by Didymin was reduced upon FoxO3a knockdown (Additional file 1: Fig. S1B). This confirms that Didymin may promote the expression of Sirt1 by activating FoxO3a.

Interestingly, molecular docking analysis indicated a physical interaction between Didymin and Sirt1 (Fig. 2J), with a complex binding energy was -78.509232 kcal/mol. The interaction sites involved were the Ala262, Ser265, Val266, Gly269, Ile270, Phe273, Arg274, Ser275, Ile347, and Asp348 (Fig. 2J). Additionally, we performed a microscale thermophoresis (MST) experiment to assess the binding of Didymin to Sirt1 protein, revealing a dissociation constant (Kd value) of 0.14683 μM (Fig. 2K). We wondered if the enzyme activity of Sirt1 could also be directly regulated by the interaction between Sirt1 and Didymin. Notably, we observed an increase in enzyme activity of purified Sirt1 recombinant protein after the addition of Didymin (about 18.92% elevated) (Fig. 2L). These findings suggest that Didymin can enhance the functionality of Sirt1 in AML12 cells by promoting Sirt1 expression and directly binding to Sirt1 protein.

In AML12 cells cultured in media containing PA, lipid accumulation was observed, as indicated by TG content and Oil Red O staining. However, administration of Didymin resulted in a reduction in hepatocellular lipid accumulation (about 73.23% reduced). Furthermore, co-treatment with EX527, a Sirt1 inhibitor, attenuated the impact of Didymin on lipid accumulation (Fig. 2M, N). Therefore, these results suggested that Didymin mitigates PA-induced lipid accumulation in AML12 cells through the Sirt1 pathway.

### Didymin improves mitochondrial biogenesis and function by activating Sirt1 in PA-treated AML12 cells

**Fig. 3:**
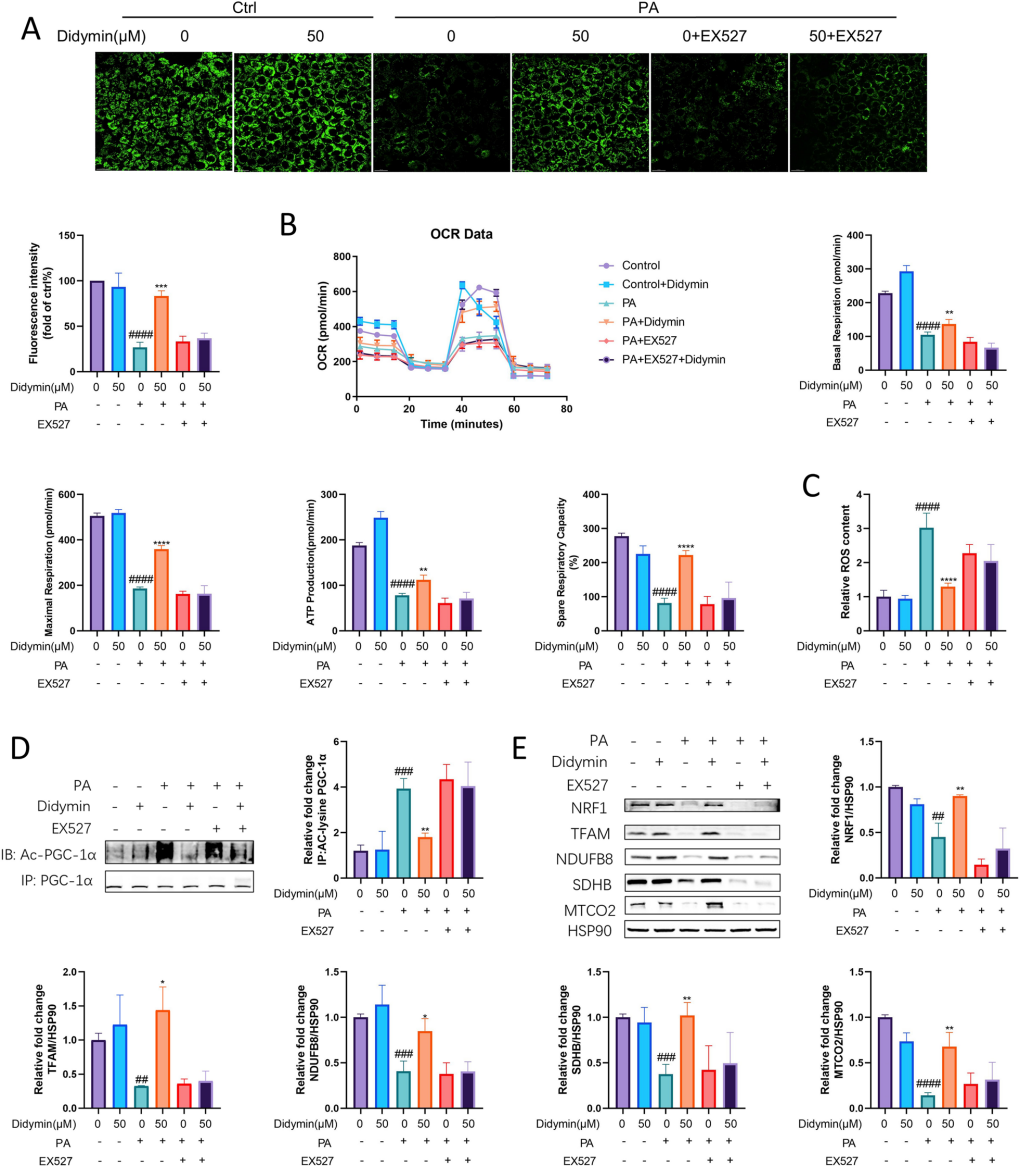
Didymin enhances mitochondrial biogenesis and function by activating Sirt1 in PA-treated AML12 cells. **A** MitoTracker Green staining for mitochondrial content in AML12 cells. (n = 3, Scale bar = 20 μm). **B** Mitochondrial oxygen consumption ratio (OCR) of AML12 cells (n = 4). **C** ROS concentration in AML12 cells (n = 4). **D** Immunoprecipitation of PGC-1α in AML12 cells showing acetylated PGC-1α level (n = 3). **E** Western blot analysis of NRF1, TFAM, NDUFB8, SDHB, and MTCO2 in AML12 cells (n = 3). Data are expressed as mean ± SD. *P < 0.05, **P < 0.01, ****P < 0.0001 PA vs. PA + Didymin. ^#^P < 0.05, ^##^P < 0.01, ^###^P < 0.001, ^####^P < 0.0001 control vs. PA. *PA* palmitic acid, *PGC-1α* proliferative activated receptor γ coactivator 1α, *NRF1* nuclear respiratory factor 1, *TFAM* mitochondrial transcription factor A

HFD-induced MAFLD is known to be associated with mitochondrial depletion and dysfunction[49], and our RNA sequencing analysis also showed that many DEGs in the PA and Didymin-treated group were associated with mitochondria function (Fig. 2E). To confirm the role of Sirt1 in regulating mitochondria function in MAFLD, we first examined the content of mitochondria in AML12 cells. The MitoTracker Green staining demonstrated that the intracellular mitochondrial content was significantly decreased in the PA-treated group compared to the control group. The treatment with Didymin increased the intracellular mitochondrial content (about 212.5% elevated), but this improvement was almost eliminated when co-treated with EX527 (Fig. 3A). To further evaluate the effect of Didymin on mitochondrial function, we measured cellular oxygen consumption rate (OCR) using Seahorse analysis. Didymin significantly reversed the PA-induced suppression of OCR, as indicated by higher levels of basal respiration, maximal respiration, ATP production, and spare respiratory capacity. However, this effect was abolished by co-treatment with EX-527 (Fig. 3B). Moreover, Didymin significantly inhibited PA-induced reactive oxygen species (ROS) production, which was further suppressed by the co-treatment with EX527 (Fig. 3C). Therefore, Didymin improves both mitochondrial content and function in PA-treated AML12 cells.

Next, we explored the underlying mechanism. Peroxisome proliferative activated receptor γ coactivator 1α (PGC-1α) is a substrate of Sirt1 deacetylation, and deacetylated PGC-1α is the biologically activated form [50]. Didymin treatment prevented the PA-induced increase in acetylated PGC-1α level (about 54.17% decreased), and co-treatment with the Sirt1-specific inhibitor EX527 nearly completely abolished the effect of Didymin, as revealed by immunoprecipitation (IP) results (Fig. 3D). Previous studies have shown that PGC-1α is a major regulator of mitochondrial biogenesis. It activates the promoter of nuclear respiratory factor 1 (NRF1), which in turn activates the promoter of mitochondrial transcription factor A (TFAM) [51]. TFAM is responsible for activating the transcription of the mitochondrial genome [52]. Western blot results showed that Didymin treatment increased the the expression of mitochondrial markers, including NRF1 and TFAM which are known to be involved in mitochondrial biogenesis, as well as genes responsible for the mitochondrial electron transport chain (ETC) such as NDUFB8, SDHB, MTCO2, in PA‐treated AML12 cells (Fig. 3E). Therefore, Didymin improves mitochondrial function in PA-treated AML12 cells through the Sirt1-PGC-1α pathway.

### Didymin reduces apoptosis and enhances lipophagy by activating Sirt1 in PA‐treated AML12 cells

**Fig. 4:**
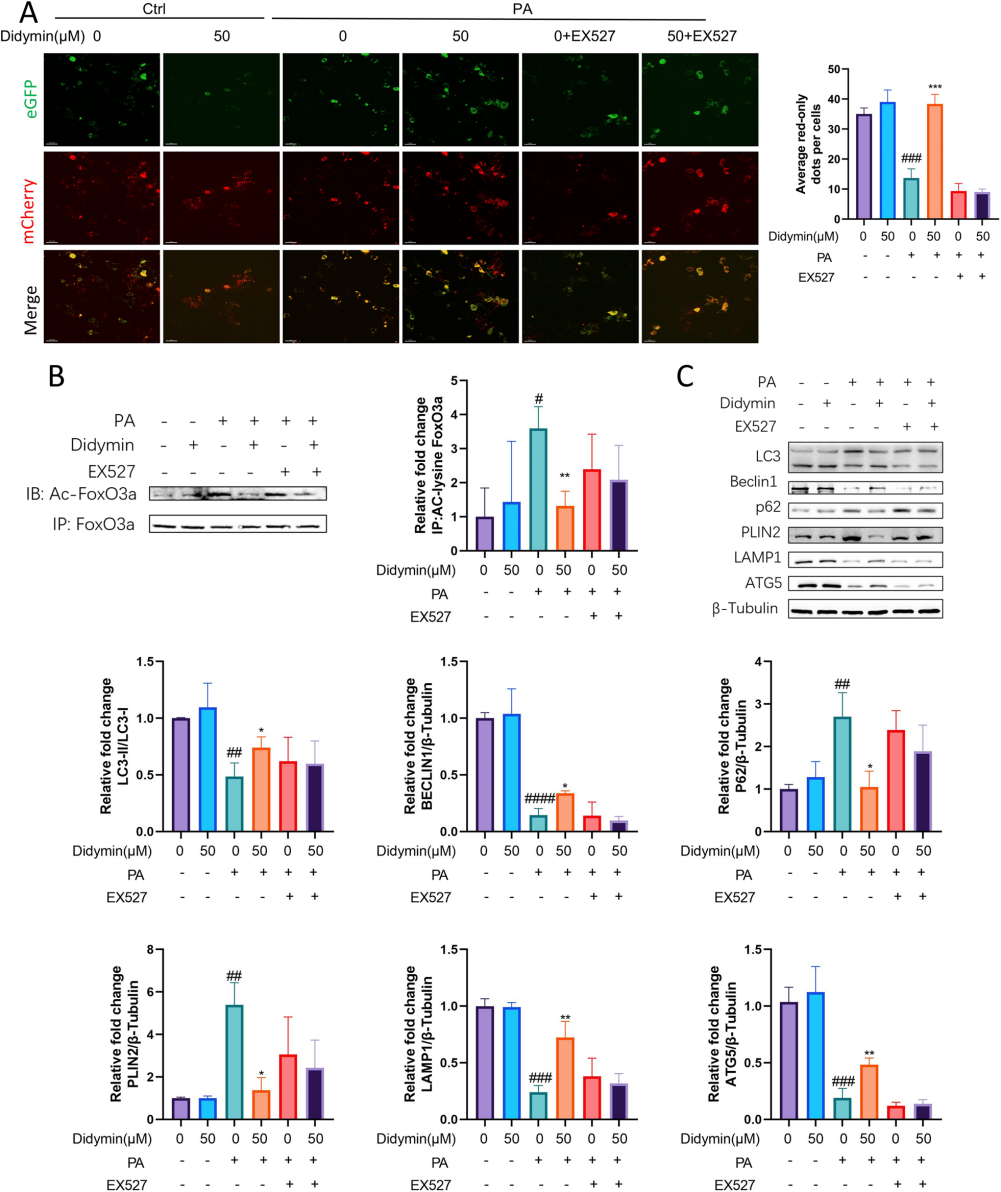
Didymin enhances lipophagy by activating Sirt1 in PA-treated AML12 cells. **A** AML12 cells transfected with mCherry-eGFP-PLIN2 plasmid. (Scale bar = 30 μm). **B** Immunoprecipitation of FoxO3a in AML12 cells showing acetylated FoxO3a level (n = 3). **C** Western blot analysis of LC3, Beclin1, P62, PLIN2, LAMP1 and ATG5 in AML12 cells (n = 3). Data are expressed as mean ± SD. *P < 0.05, **P < 0.01 PA vs. PA + Didymin. ^##^P < 0.01, ^###^P < 0.001, ^####^P < 0.0001 control vs. PA. *PA* palmitic acid, *FoxO3a* Foxkhead Box Class O 3a, *PLIN2* perilipin 2, *LAMP1* lysosomal associated membrane protein 1

Elevated free fatty acids (FFAs) lead to the accumulation of LDs in hepatocytes [53], and autophagy plays a crucial role in the breakdown of these LDs, a process known as lipophagy [54]. Lipotoxicity induced by a high-fat diet impairs the lipophagy process and promotes lipid accumulation [55]. Autophagy is also important in eliminating damaged organelles and degraded protein from hepatocytes [46]. Apoptotic and autophagic signaling pathways are interconnected, and both contribute to hepatocyte function, while autophagy deficiency is related with an accumulation of cell damage and an increase in cell death[56]. Our sequencing results showed that the autophagy pathway is regulated by Didymin (Fig. 2C, D). After the addition of Didymin, the expression levels of some autophagy-related genes decreased (Fig. 2F). We further analyzed the expression of lipid autophagy-related proteins and found that the expression of some lipophagy-related genes increased, while the protein expression of lipid droplet surface (PLIN2 and PLIN3) decreased (Additional file 1: Fig. S2).To further investigate the roles of Didymin in these processes, we generated a mCherry-GFP-PLIN2 fusion protein to monitor the lysosomal degradation of LDs. The intracellular red fluorescence represents the process of lipophagy. The results showed that Didymin rescued the lipophagy, as evidenced by elevation in the number of red puncta (about 180.49% elevated) (Fig. 4A). Moreover, the inhibition of Sirt1 by EX-527 treatment blocked the lipophagy of AML12 cells (Fig. 4A). These results indicate that Didymin enhances lipophagy by activating Sirt1 in PA-treated AML12 cells, consistent with the previous studies highlighting the role of Sirt1 deacetylase as a key regulator of autophagy [57].

Previous work has shown that Sirt1 enhances FoxO3a deacetylation, which increases FoxO3a-mediated transcription of Autophagy Protein (Atg) genes [58]. Notably, we found that Didymin restored the PA-induced decrease in acetylated FoxO3a level (about 63.48% decreased), whereas this effect was abolished by co-treatment with EX527 (Fig. 4B). To examine the mechanism of Didymin in lipophagy, we detected the expression levels of lipophagy-related proteins, such as microtubule-associated protein light chain 3 (LC3), Beclin1, p62, and ATG5, as well as LDs and lysosomal membrane markers PLIN2 and LAMP1. Lipophagy was decreased by PA administration when compared to the control group, as evidenced by a lower LC3II/LC3I ratio, lower Beclin1 expression, and higher p62 expression (Fig. 4C). Furthermore, these effects were partially reversed by the treatment of Didymin, while the effects of Didymin can be abolished by EX-527. Overall, Didymin regulates lipophagy through Sirt1-FoxO3a.

**Fig. 5:**
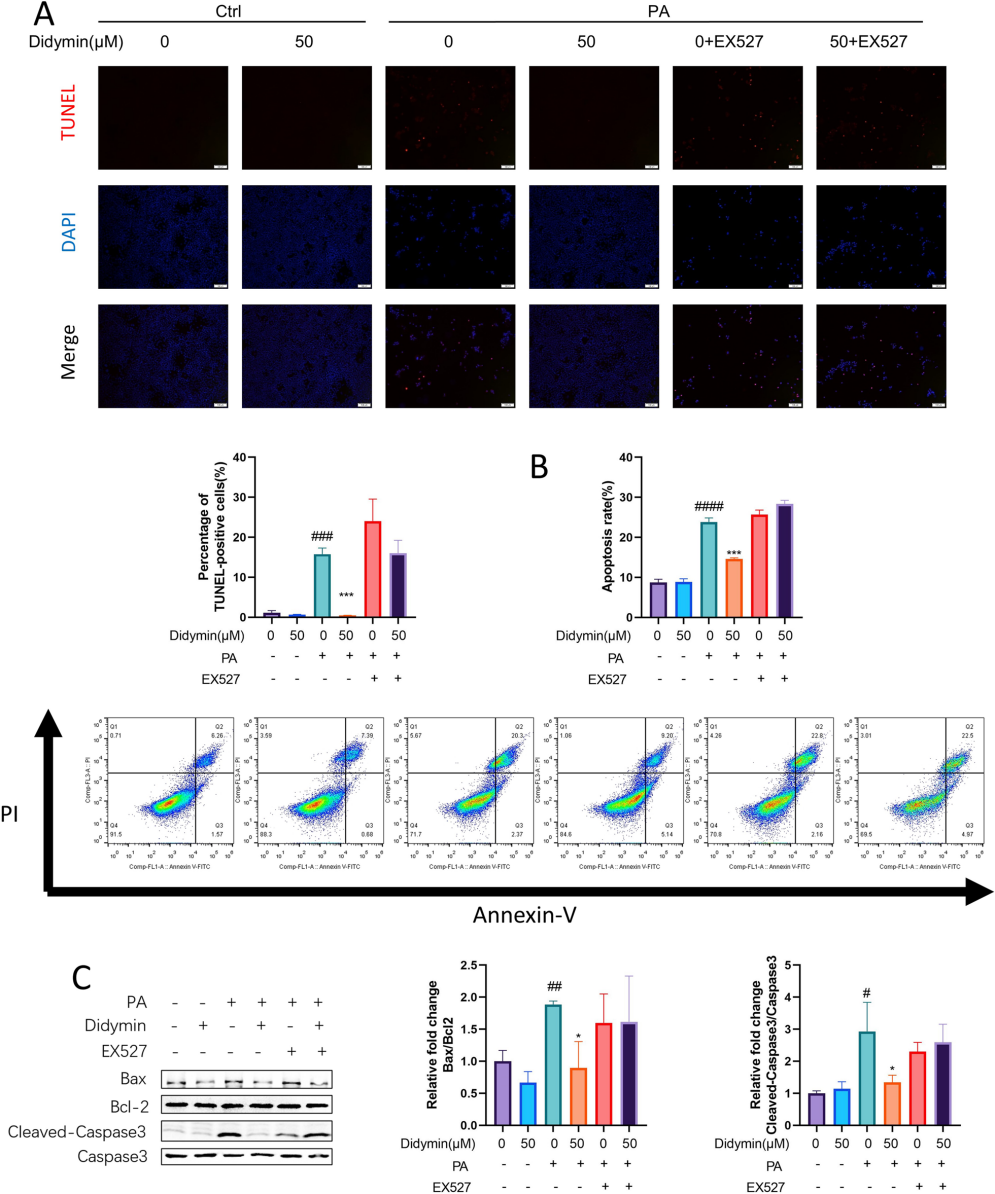
Didymin suppresses apoptosis by activating Sirt1 in PA-treated AML12 cells. **A** Representative images of TUNEL staining in AML12 cells (Scale bar = 100 μm). Quantification of the percentage of TUNEL-positive cells (n = 3). **B** Apoptosis analysis of AML12 cells by flow cytometry. And the results of quantitative analyses of apoptosis rate (n = 3). **C** Western blot analysis of Bax, Bcl2, cleaved-caspase3, and caspase3 proteins in AML12 cells (n = 3). Data are expressed as mean ± SD. *P < 0.05, ***P < 0.001 PA vs. PA + Didymin. # P < 0.05, ## P < 0.01, ### P < 0.001, #### P < 0.0001 control vs. PA. *PA* palmitic acid, *TUNEL* TdT-mediated dUTP Nick-End labeling, *Bax* Bcl-2-associated X protein, *Bcl2* B-cell lymphoma 2

According to the RNA sequencing analysis, apoptosis might also be involved in the process of Didymin in PA-treated AML12 cells (Fig. 2G). TUNEL assay results showed that the number of apoptotic cells significantly increased after PA treatment and was down-regulated by Didymin (about 97.25% decreased). However, co-treatment of Didymin and EX-527 resulted in a re-increase in the number of apoptotic cells (Fig. 5A). Flow cytometry analysis supported these findings (Fig. 5B). Bcl-2-associated X protein (Bax), B-cell lymphoma 2 (Bcl2), and Caspase3 were critical components of the apoptotic process. According to western blot analysis, Cleaved-Caspase3/Caspase3 and Bax/Bcl-2 ratios were reduced by PA but were recovered after the addition of Didymin. However, the improvement was inhibited by the co-treatment of Didymin and EX-527 (Fig. 5C). Thus, Didymin suppresses apoptosis stimulated by PA in AML12 cells by activating Sirt1.

Autophagy dysfunction contributes to the aggregation of LDs and misfolded proteins, ultimately leading to cell degeneration[56]. We used the autophagy inhibitor 3-Methyladenine (3-MA, 1 mM) to confirm whether Didymin suppresses apoptosis by restoring lipophagy in PA-treated AML12 cells. As expected, western blot results indicated that Didymin improved the reduction in the Cleaved-Caspase3/Caspase3 and Bax/Bcl-2 ratios caused by PA treatment (Additional file 1: Fig. S3A). However, co-treatment of autophagy inhibitor 3-MA nearly abolished the impact of Didymin on apoptosis (Additional file 1: Fig. S3A). We also performed the TUNEL assay (Additional file 1: Fig. S3B) and the flow cytometry analysis (Additional file 1: Fig. S3C) to measure cell apoptosis, and the results were consistent with the western blot results. Overall, Didymin alleviates PA-induced apoptosis in AML12 cells by activating autophagy to restore cellular homeostasis.

### Didymin protects mice against high-fat diet-induced MAFLD by activating Sirt1

**Fig. 6:**
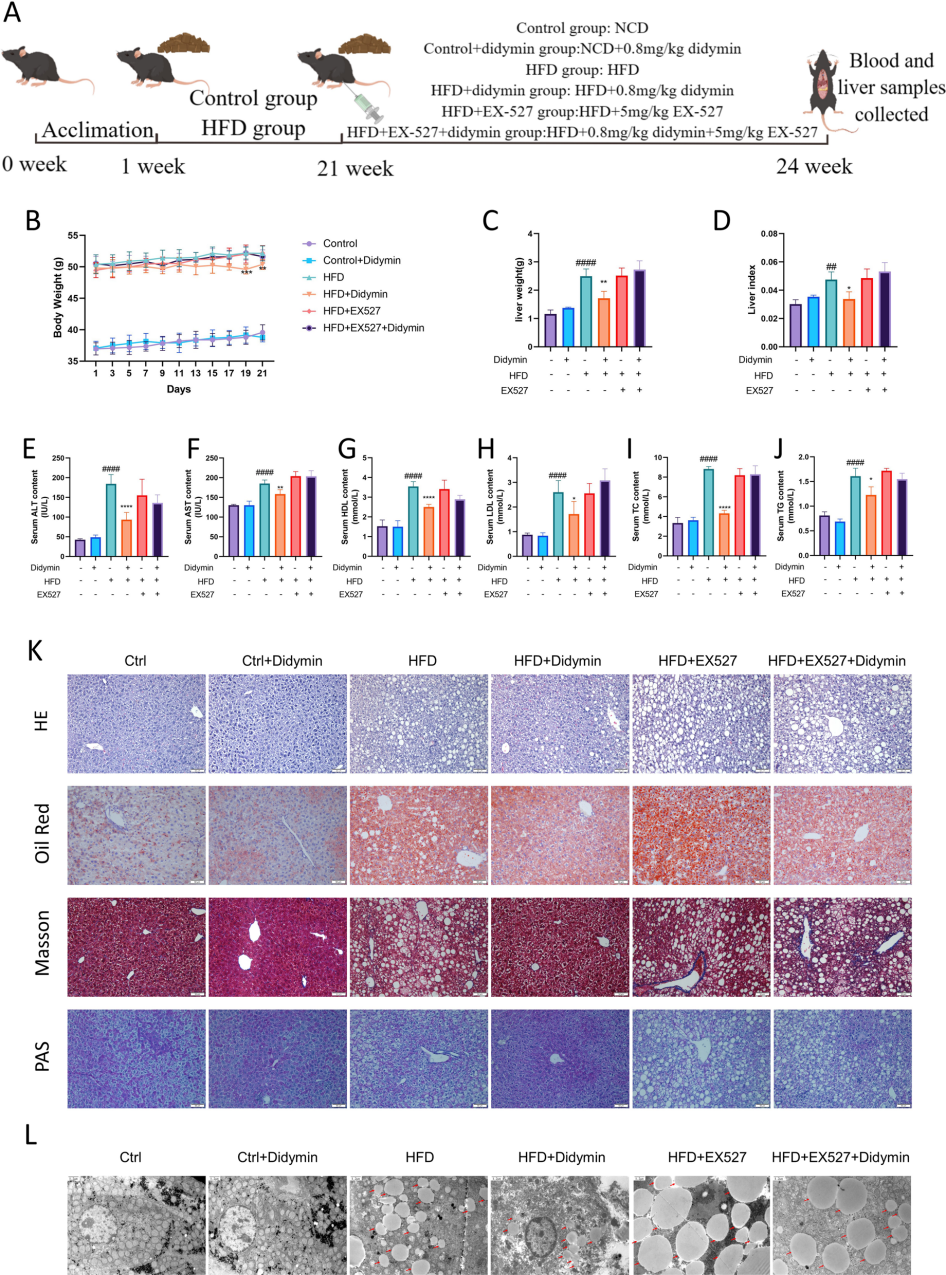
Didymin protects mice against high-fat diet-induced MAFLD by activating Sirt1. **A** Graphical description of the experimental design of this study. **B** Body weight (n = 6–8). **C** Liver weight. **D** Liver index. **E–J** Serum level of TG, TC, ALT, AST, LDL and HDL (n = 5–6). **K** Representative H&E, oil red O, Masson, and PAS staining of hepatic sections (Scale bar = 50 μm). **L** Transmission electron microscopy figures of hepatocytes, arrows show lipid droplets (Scale bar = 1.2 μm). All values were expressed as the mean ± SD. *P < 0.05, **P < 0.01, ****P < 0.0001 PA vs. PA + Didymin. ^##^P < 0.01, ^####^P < 0.0001 control vs. MAFLD. *PA* palmitic acid, *MAFLD* metabolic associated fatty liver disease, *TG* triglyceride, *TC* total cholesterol, *ALT* alanine aminotransferase, *AST* aspartate aminotransferase, *LDL* low-density lipoprotein, *HDL* high-density lipoprotein, *H&E* hematoxylin, and eosin

To validate the roles and underlying mechanisms of Didymin in hepatocytes, we further conducted in vivo assays (Fig. 6A). The weight of the body and liver decreased (about 31.23%) after Didymin administration in MAFLD mice, but the effect was reversed by concurrent treatment with Didymin and EX-527 (Fig. 6B–D). The liver specimens of the control group exhibited a typical liver morphology characterized by a reddish-brown color, well-defined borders, and a smooth surface, while the MAFLD group showed pathological symptoms such as a yellow appearance, rough and granular surface, a blunt edge, and a larger size. Didymin significantly reduced these pathological symptoms, but co-treatment with EX-527 eliminated the effect (Additional file 1: Fig. S4A). Didymin also reduced serum levels of ALT (about 49.34%), AST (about 14.30%), HDL (about 29.44%), LDL (about 34.13%), TC (about 50.90%), and TG (about 23.59%) in HFD-induced MAFLD mice, but these effects were abolished by co-treatment with EX-527 (Fig. 6E–J). Didymin alleviated ballooning degeneration, lipid accumulation, and fibrosis, and enhanced glycogen content in the livers of MAFLD mice, as indicated by H&E, oil red O, Masson, and PAS staining (Fig. 6K). The transmission electron microscopy images of hepatocytes also showed LD accumulation, consistent with the results of oil red O staining (Fig. 6L).

**Fig. 7:**
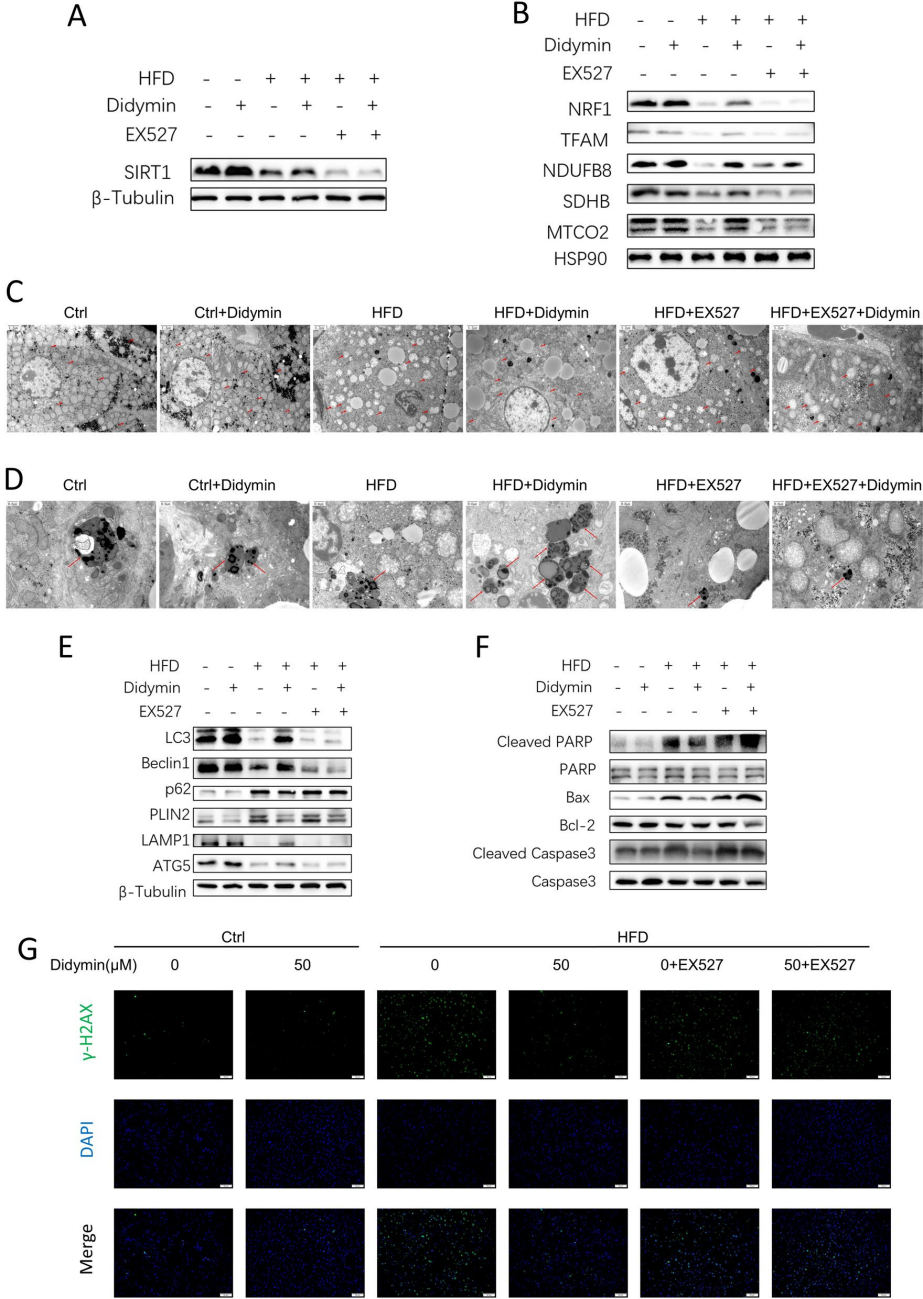
Didymin enhances mitochondrial biogenesis and function, promotes lipophagy, and inhibits apoptosis by activating Sirt1 in mice hepatocytes. **A** Western blot analysis of Sirt1 expression in hepatocytes (n = 3). **B** Western blot analysis of NRF1, TFAM, NDUFB8, SDHB, and MTCO2 in hepatocytes. **C** Transmission electron microscopy figures of hepatocytes, arrows show mitochondria (Scale bar = 0.6 μm). **D** Transmission electron microscopy figures of hepatocytes, arrows show autolysosomes (Scale bar = 0.6 μm). **E** Western blot analysis of LC3, Beclin1, P62, PLIN2, LAMP1, and ATG5 in hepatocytes (n = 3). **F** Western blot analysis of Cleaved PARP, Bax, Bcl2, Cleaved Caspase 3, and Caspase 3 proteins in hepatocytes (n = 3). **G** Immunofluorescence staining of γ-H2AX in hepatocytes (Scale bar = 50 μm). *NRF1* nuclear respiratory factor 1, *TFAM* mitochondrial transcription factor A, *PLIN2* perilipin 2, *LAMP1* lysosomal associated membrane protein 1, *Bax* Bcl-2-associated X protein, *Bcl2* B-cell lymphoma 2

Furthermore, treatment of Didymin eliminated the decrease in expression of Sirt1, PGC-1α, and FoxO3a in the livers of MAFLD mice. However, when co-treatment with EX-527, the effects were eradicated (Fig. 7A, Additional file 1: Fig. S5). Didymin also increased the expression of NRF1, TFAM, NDUFB8, SDHB, and MTCO2 in the livers of MAFLD mice by activating the Sirt1-PGC-1α pathway, while these improvements were almost thwarted by cotreatment with EX527 and Didymin (Fig. 7B). Transmission electron microscopy images of MAFLD mouse hepatocytes indicated mitochondrial structural damage, including ballooning, shallower matrix, and shorter and fewer or even disappeared cristae. Administration of Didymin treatment relieved these damages, but this improving effect was abrogated again by co-administration with EX-527 and Didymin (Fig. 7C). Didymin treatment increased the amount of autolysosome in MAFLD mice hepatocytes, but co-treatment with EX-527 abolished its effect (Fig. 7D). Didymin-induced lipophagy in hepatocytes of MAFLD mice was also indicated by a higher LC3-II/LC3-I ratio, higher expression levels of Beclin1 and ATG5, as well as lower p62 expression (Fig. 7E). Higher LAMP1 and lower PLIN2 protein levels also indicated the activation of lipophagy in Didymin-treated MAFLD mice hepatocytes (Fig. 7E). Moreover, Didymin suppressed the ratios of cleaved PARP/PARP, cleaved caspase3/caspase3, and Bax/Bcl-2, indicating inhibited apoptosis (Fig. 7F). The γ-H2AX immunofluorescence stain results also indicated the same findings (Fig. 7G).

**Fig. 8:**
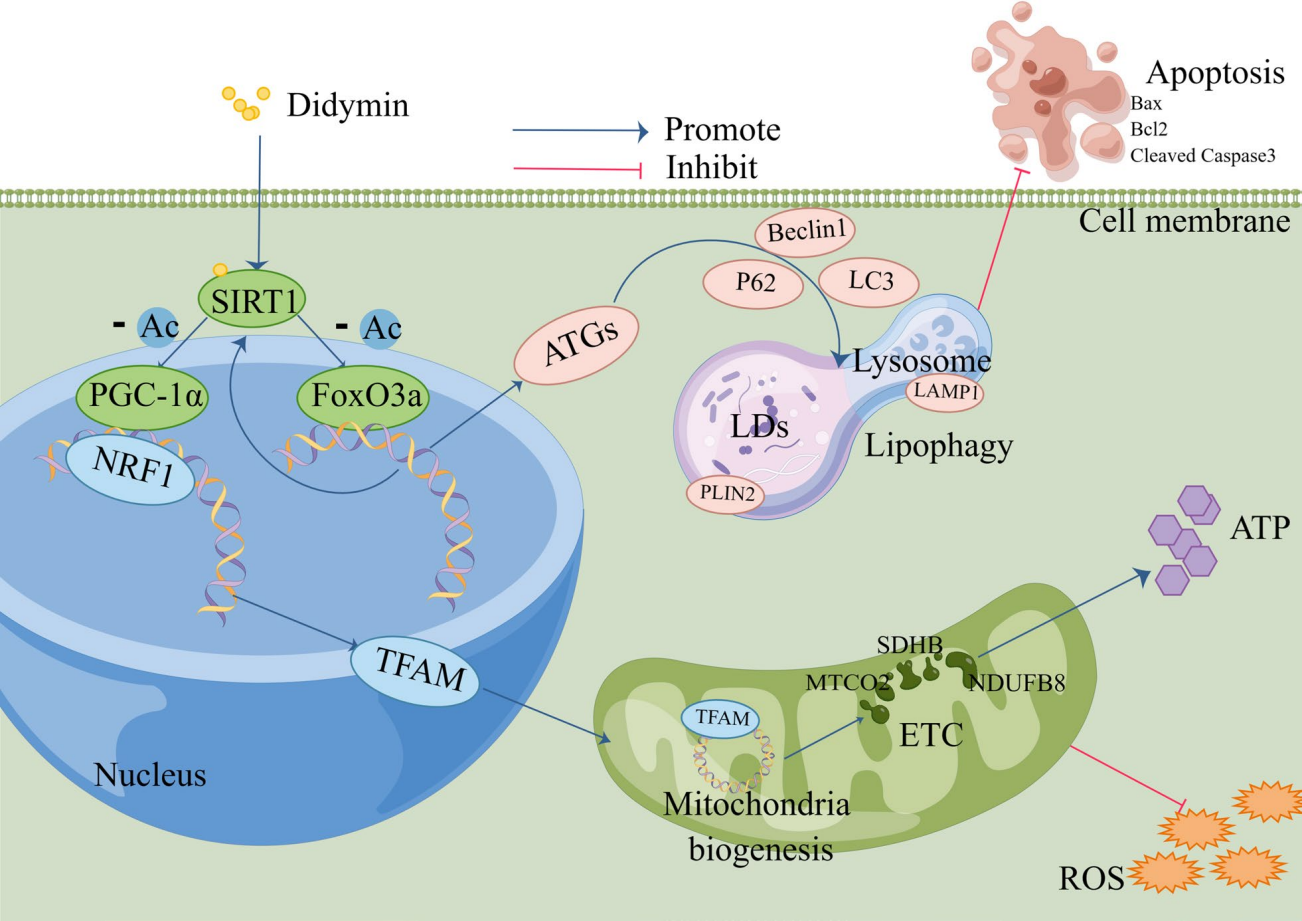
Schematic representation of Didymin alleviates MAFLD through the Sirt1 pathway. Didymin binds to SIRT1 protein and activates its deacetylase activity, which in turn deacetylates FoxO3a and PGC-1α and enhances their activity. FoxO3a further increases the expression level of SIRT1 and promotes the process of lipophagy. The increase in lipophagy activity can further inhibit cell apoptosis. PGC-1α enhances mitochondrial biosynthesis and improves mitochondrial function by promoting the transcription of NRF1 and TFAM. *PGC-1α* proliferative activated receptor γ coactivator 1α, *NRF1* nuclear respiratory factor 1, *TFAM* mitochondrial transcription factor A, *FoxO3a* Foxkhead Box Class O 3a, *PLIN2* perilipin 2, *LAMP1* lysosomal associated membrane protein 1, *Bax* Bcl-2-associated X protein, *Bcl2* B-cell lymphoma 2

In conclusion, Didymin protects against high-fat-induced MAFLD through the Sirt1 pathway, promoting mitochondrial biogenesis and function, enhancing lipophagy, and inhibiting apoptosis (Fig. 8).

## Discussion

This is the first study to show that Didymin alleviates MALFD by activating Sirt1 in both high-fat driven AML12 cells and MAFLD animals. Didymin improves mitochondrial biogenesis and function, as well as augmentes lipophage and attenuates apoptosis via the Sirt1-PGC-1α/FoxO3a pathway. Therefore, Didymin may be a potential treatment option for MAFLD. Additionally, Didymin was identified as a novel Sirt1 activator.

The presence of MAFLD is correlated with obesity and is present in up to 91% of severely obese patients. High-fat diet intake allows animals to develop obesity, hyperinsulinemia, hyperglycemia, hypertension, and liver damage, similar to the phenotype observed in humans with MAFLD [59]. Therefore, the high-fat diet-induced mouse model of MAFLD is one of the most commonly used animal models of MAFLD [60–62]. In experiments comparing male and female mice on a high-fat diet, male mice are more likely to experience weight gain [63–65], which may be caused by differences in estrogen levels [66], sex-specific leptin resistance [67, 68], and gross motor activity [64]. Therefore, almost all the experiments using high-fat diet to construct animal models of MAFLD were conducted in male mice [69].

Initially, we observed that Didymin decreased lipid accumulation induced by high fat in both in vivo and in vitro models, as evidenced by using oil red O staining. Further analysis of intracellular TG levels and cell survival in AML12 cells confirmed the therapeutic effect of Didymin on MAFLD (Fig. 1). Excessive nutrient intake leads to MAFLD, where most ingested fat is directed to adipose tissue or working muscles for storage or oxidation. However, fat stored in white adipose tissue undergoes lipolysis to release fatty acids. Excess fatty acids result in TG accumulation in hepatocytes, leading to lipotoxicity and impaired cell function [4]. Hepatic steatosis is an important diagnostic index for MAFLD, and reducing hepatocyte steatosis is crucial for its treatment [70].

To elucidate the underlying mechanism of Didymin's therapeutic effect on MAFLD, we conducted a comprehensive investigation based on RNA sequencing data. The results indicate that Didymin mainly affected the autophagy, apoptosis, and mitochondria pathways, all of which are intricately associated with the involvement of Sirt1 (Fig. 2). Sirt1 plays a critical role in regulating hepatocyte metabolism [71], by directly deacetylating and activating PGC-1α to promote mitochondrial biogenesis [72] and modifying FoxOs transcriptional activity to regulate autophagy [73]. Therefore, activating Sirt1 may be a promising therapeutic strategy for treating MAFLD [74], as demonstrated by the efficacy of other Sirt1 activators such as metformin, liraglutide, and antrodan [75–77].

We observed that treatment with Didymin resulted in the upregulation of Sirt1 expression and enhanced the deacetylase activity of Sirt1. Interestingly, we found that Didymin could enhance Sirt1's deacetylase activity through direct binding (Fig. 2). Previous studies have demonstrated that Sirt1 directly deacetylates PGC-1α and FoxOs, thereby regulating their nuclear translocation and activity [73, 78]. To further investigate this, we evaluated Sirt1's deacetylase activity as well as the acetylation levels of PGC-1α and FoxO3a in cells treated with Didymin and a Sirt1 inhibitor. Our findings suggested that Didymin activates the Sirt1-PGC-1α/FoxO3a pathway in both mouse hepatocytes and AML12 cells.

FoxO3a plays a critical role in regulating hepatocellular autophagy [58] and can modulate the transcription of autophagy-related genes [79]. Our study revealed that Didymin treatment increased Sirt1 and FoxO3a expression, which in turn prompted autophagy in the context of lipotoxicity-induced damage. Furthermore, Sirt1 inhibition eliminated the protective effect of Didymin against lipotoxicity (Figs. 4, 7). These results highlighted the crucial role of the Sirt1/ FoxO3a pathway in mediating the protective effects of Didymin. Maintaining optimal autophagy levels aids liver cells in eliminating lipid droplets (also known as lipophagy), damaged organelles, and wasted proteins [80, 81]. The lipotoxicity-induced suppression of autophagy accelerates the accumulation of lipid droplets in liver cells and triggers hepatocellular apoptosis [82, 83]. Activation of FoxO3a can induce autophagy and improve liver cell function [84–86].

Our experimental results indicate that Didymin promotes lipophagy in hepatocytes. However, in our initial sequencing results, although it suggests that cell autophagy is regulated by Didymin, the expression of many genes is downregulated, which seems to suggest that Didymin may inhibit the autophagy process. Therefore, we further analyzed the expression of genes involved in lipophagy in the sequencing data and found that the expression of some genes increased. Meanwhile, studies have shown that autophagy activity is not only influenced by protein levels but also post-transcriptionally regulated, such as acetylation, phosphorylation, etc. [87, 88]. Therefore, we believe that although the expression of some autophagy-related proteins decreases, the overall level of cellular autophagy is still increased due to the regulation of post-translational modifications and the upregulation of other protein expressions. We will further explore the molecular mechanisms by which Didymin regulates the process of cellular autophagy.

To examine the effect of Didymin on apoptosis, we utilized TUNEL labeling, flow cytometry, and Western blotting to detect damaged DNA strands, phosphatidylserine flipping, and levels of apoptotic key proteins, respectively. Didymin was found to decrease apoptosis by restoring Sirt1-mediated autophagy in PA-treated AML12 cells, and this effect was abolished by adding the Sirt1 inhibitor (EX-527) or the autophagy inhibitor (3MA) (Figs. 5, 7, Additional file 1: Fig. S2). These findings indicated that Didymin's protection against MAFLD was mediated through the regulation of autophagic and apoptotic signaling pathways.

Furthermore, we evaluated mitochondria content, ROS concentration, and the functionality of mitochondrial ETC with or without the treatments of Didymin or Sirt1 inhibitor. These results demonstrated that Didymin improved mitochondrial biogenesis and function by activating Sirt1. Although Didymin did not directly alleviate the lipid deposition in hepatocytes, improving mitochondrial function can regulate lipid metabolism and alleviate oxidative stress-induced damage, which are also critical for ameliorating MAFLD [89]. Recent studies have also shown that stimulation of mitochondrial biogenesis by indole-3-acetic acid or L-Carnitine via the PGC-1α pathway leads to enhanced mitochondrial function and alleviates MAFLD [90, 91].

We also included another SIRT1 inhibitor, SIRT1-IN-1, for validation. The results also showed that after inhibiting the activity of SIRT1, Didymin was unable to exert its improvement on lipid deposition (Additional file 1: Fig. S6A), mitochondrial biogenesis (Additional file 1: Fig. S6B), and lipophagy (Additional file 1: Fig. S6C) in AML12 cells. These further proved that Didymin exerts therapeutic effects through SIRT1.

Didymin reduces hepatic lipid deposition, lowers plasma lipid levels, and decreases hepatocyte damage. Due to the current lack of drugs specifically targeting MAFLD, Didymin has the potential to be used as an adjunctive therapy for treating MAFLD and lowering plasma lipid levels. The activation of SIRT1 by Didymin suggests its potential therapeutic effects in other mitochondrial and autophagy-related diseases, promoting mitochondrial protection and autophagy. In addition to improving lipid deposition in liver cells, another significant therapeutic effect of Didymin is the reduction of serum fatty acids levels. Our research shows that the increased lipophagy induced by Didymin promotes the decomposition of LDs into FFAs. At the same time, the improvement in mitochondrial function allows for more fatty acids to be oxidized by mitochondrial fatty acid oxidation, thereby reducing the serum levels of fatty acids. The other main way to reduce serum FFAs levels is to break down and utilize them by muscle and adipose tissues [92]. We have not yet explored the effect of Didymin on the metabolism of these organs, and this will be the direction of our future research.

Previous studies have shown the therapeutic effects of Didymin on liver insulin resistance in diabetes [93]. This study discovered the binding and regulatory effects of Didymin on key proteins in the insulin signaling pathway, demonstrating its improvement on oxidative stress and insulin resistance in liver cells. Although this study mainly focused on the insulin resistance model in type 2 diabetes, it also indicated its beneficial effects on oxidative stress. Another study demonstrated the therapeutic effects of Didymin on liver fibrosis and acute liver injury, suggesting its protective effects on oxidative stress and apoptosis in liver cells [94, 95]. These findings suggest that although the pathways involved may differ, they all indicate the protective effects of Didymin on the liver in different disease models. A study on the therapeutic effects of Naringenin on NASH [96] showed that Naringenin improves hepatic steatosis, hepatic fibrosis, hepatic inflammation, and hepatic oxidative stress in NASH mice, and it suggests that Naringenin exerts its therapeutic effects through SIRT1. Due to the similar chemical structure of flavonoid glycosides, the activation of SIRT1 by Naringenin also supports our research finding that SIRT1 is the target of another flavonoid glycoside compound Didymin. Furthermore, several studies on other flavonoid glycoside compounds such as Hesperetin [97], Wogonin [98], Myricetin [99], and swertiamarin [100] have also demonstrated the improvement of hepatic lipid deposition through various mechanisms such as oxidative stress, autophagy, and lipid metabolism.

This study comprehensively analyzed the therapeutic effects of Didymin on MAFLD through in vivo and in vitro experiments. The role of Didymin was fully validated as a target through molecular experiments and in vivo and in vitro experiments with the addition of SIRT1 inhibitors. However, there is still a lack of detection regarding its metabolic improvements on other organs or side effects. Also, although no significant toxicity of Didymin has been shown in the existing studies and this study, there is still a lack of research on its side effects. Therefore, future studies will focus on fully elucidating the molecular mechanism of Didymin in the treatment of MAFLD and other metabolic diseases.

## Conclusions

Collectively, our current findings demonstrate that Didymin, identified as a specific activator of Sirt1, ameliorates MAFLD through promoting mitochondrial biogenesis and function, enhancing lipophagy, and inhibiting apoptosis (Fig. 8). These will contribute to the development of therapeutic strategies for MAFLD. Didymin, as a Sirt1 agonist, may also have therapeutic potential in treating other diseases.

### Supplementary Information

**Additional file 1:** **Figure S1.** Didymin promotes the expression of Sirt1 by activating FoxO3a. (A) Western blot analysis of FoxO3a proteins in AML12 cells after siRNA transfection (n=3). (B) Western blot analysis of Sirt1 and FoxO3a proteins in AML12 cells after siRNA transfection (n=3). Data are expressed as mean ± SD. *P < 0.05, **P < 0.01. **Figure S2.** Heatmap of lipophagy-related genes. The red boxes indicate proteins directly regulated by FoxO3a, histone H4 K16, and TFEB. **Figure S3. **Didymin suppresses apoptosis by restoring lipophagy in PA-treated AML12 cells. (A) Representative images of TUNEL staining in AML12 cells (Scale bar = 100 μm). Quantification of the percentage of TUNEL-positive cells (n=3). (B) Apoptosis analysis of AML12 cells by flow cytometry. The results of quantitative analyses of apoptosis rate (n=3). (C) Western blot analysis of Bax, Bcl2, cleaved-caspase3, and caspase3 proteins in AML12 cells (n = 3). Data are expressed as mean ± SD. *P < 0.05, ***P < 0.001 PA vs. PA+Didymin. # P < 0.05, ## P < 0.01, ### P < 0.001, #### P < 0.0001 control vs. PA. **Figure S4. **Liver morphology and statistical analysis of western blot results. (A) Liver morphology (B) Statistical analysis of western blot results in Figure 7A (n=3). (C) Statistical analysis of western blot results in Figure 7B (n=3). (D) Statistical analysis of western blot results in Figure 7E (n=3). (E) Statistical analysis of western blot results in Figure 7F (n=3). Data are expressed as mean ± SD. *P < 0.05, **P < 0.01 MAFLD vs. MAFLD+Didymin. # P < 0.05, ## P < 0.01, ### P < 0.001, #### P < 0.0001 control vs. MAFLD. **Figure S5. **Western blot analysis of PGC-1α and FoxO3a in hepatocytes (n=3). Data are expressed as mean ± SD. *P < 0.05, **P < 0.01 MAFLD vs. MAFLD+Didymin. # P < 0.05, ## P < 0.01 control vs. MAFLD. **Figure S6. **Sirt1-in-1 inhibits the activation of SIRT1 by Didymin in AML12 cells. (A) TG contents in AML12 cells (n=4). (B) Western blot analysis of NRF1 and TFAM in hepatocytes (n=3). (C) Western blot analysis of Beclin1, p62, and LAMP1 in hepatocytes (n=3). Data are expressed as mean ± SD. **P < 0.01, ***P < 0.001, ****P< 0.0001 PA vs. PA+Didymin. ## P < 0.01, ### P < 0.001, #### P < 0.0001 control vs. PA. **Figure S7.** Cell viability of AML12 cells treated with PA and different concentrations of Didymin (n=4). Data are expressed as mean ± SD. ****P< 0.0001 PA vs. PA+Didymin.

## Data Availability

The crystallographic structure of Sirt1 utilized in this investigation was acquired from Brookhaven Protein Data Bank (PDB entry: 4ZZH). The structure of Didymin was obtained from the ChemSpider database (ChemSpider ID: 16498764). All data are available from the corresponding author upon reasonable request.
