# Supplementary material for: Didymin alleviates metabolic dysfunction-associated fatty liver disease (MAFLD) via the stimulation of Sirt1-mediated lipophagy and mitochondrial biogenesis
Source: J Transl Med. 2023 Dec 19;21:921. doi: 10.1186/s12967-023-04790-4 (PMC10731721; doi:10.1186/s12967-023-04790-4)
Supplement: Supplementary file 1 — Additional file 1: Figure S1. Didymin promotes the expression of Sirt1 by activating FoxO3a. (A) Western blot analysis of FoxO3a proteins in AML12 cells after siRNA transfection (n=3). (B) Western blot analysis of Sirt1 and FoxO3a proteins in AML12 cells after siRNA transfection (n=3). Data are expressed as mean ± SD. *P < 0.05, **P < 0.01. Figure S2. Heatmap of lipophagy-related genes. The red boxes indicate proteins directly regulated by FoxO3a, histone H4 K16, and TFEB. Figure S3. Didymin suppresses apoptosis by restoring lipophagy in PA-treated AML12 cells. (A) Representative images of TUNEL staining in AML12 cells (Scale bar = 100 μm). Quantification of the percentage of TUNEL-positive cells (n=3). (B) Apoptosis analysis of AML12 cells by flow cytometry. The results of quantitative analyses of apoptosis rate (n=3). (C) Western blot analysis of Bax, Bcl2, cleaved-caspase3, and caspase3 proteins in AML12 cells (n = 3). Data are expressed as mean ± SD. *P < 0.05, ***P < 0.001 PA vs. PA+Didymin. # P < 0.05, ## P < 0.01, ### P < 0.001, #### P < 0.0001 control vs. PA. Figure S4. Liver morphology and statistical analysis of western blot results. (A) Liver morphology (B) Statistical analysis of western blot results in Figure 7A (n=3). (C) Statistical analysis of western blot results in Figure 7B (n=3). (D) Statistical analysis of western blot results in Figure 7E (n=3). (E) Statistical analysis of western blot results in Figure 7F (n=3). Data are expressed as mean ± SD. *P < 0.05, **P < 0.01 MAFLD vs. MAFLD+Didymin. # P < 0.05, ## P < 0.01, ### P < 0.001, #### P < 0.0001 control vs. MAFLD. Figure S5. Western blot analysis of PGC-1α and FoxO3a in hepatocytes (n=3). Data are expressed as mean ± SD. *P < 0.05, **P < 0.01 MAFLD vs. MAFLD+Didymin. # P < 0.05, ## P < 0.01 control vs. MAFLD. Figure S6. Sirt1-in-1 inhibits the activation of SIRT1 by Didymin in AML12 cells. (A) TG contents in AML12 cells (n=4). (B) Western blot analysis of NRF1 and TFAM in hepatocytes (n=3). (C [file 12967_2023_4790_MOESM1_ESM.docx]

**Methods:**

**Library construction, sequencing, and data analysis of RNA-seq:**

Sample QC: Select the corresponding testing methods for quality inspection according to the requirements of samples and products.

mRNA Isolation: A certain amount of RNA samples are denatured at suitable temperature to open their secondary structure, and mRNA is enriched by oligo (dT) -attached magnetic beads.

mRNA Fragmentation: The reaction system is configured. After reacting at the suitable temperature for a fixed period of time, RNAs are fragmented.

cDNA Synthesis: Prepare the first-strand synthesis reaction system, and set up the reaction program, synthesize the first- strand cDNA, prepare the second-strand synthesis reaction system, and set up the reaction program to synthesize the second-strand cDNA.

End Repair, Add A and Adaptor Ligation: After the reaction system and program are configured and set up, double-stranded cDNA fragments are subjected to end-repair, and then a single ‘A’ nucleotide is added to the 3’ ends of the blunt fragments. The reaction system and program for adaptor ligation are subsequently configured and set up to ligate adaptors with the cDNAs.

PCR: The PCR reaction system and program are configured and set up to amplify the product.

Library QC: The corresponding library quality control protocol will be selected depending upon product requirements.

Circularization: Single-stranded PCR products are produced via denaturation. The reaction system and program for circularization are subsequently configured and set up. Single-stranded cyclized products are produced, while uncyclized linear DNA molecules are digested.

Sequencing: Single-stranded circle DNA molecules are replicated via rolling cycle amplification, and a DNA nanoball (DNB) which contain multiple copies of DNA is generated. Sufficient quality DNBs are then loaded into patterned nanoarrays using high-intensity DNA nanochip technique and sequenced through combinatorial Probe-Anchor Synthesis (cPAS).

Data filtering: The sequencing data was filtered with SOAPnuke [1] by (1) Removing reads containing sequencing adapter; (2) Removing reads whose low-quality base ratio (base quality less than or equal to 15) is more than 20%; (3) Removing reads whose unknown base ('N' base) ratio is more than 5%, afterwards clean reads were obtained and stored in FASTQ format. The subsequent analysis and data mining were performed on Dr. Tom Multi-omics Data mining system (https://biosys.bgi.com).

RNA identification: Bowtie2[2] was applied to align the clean reads to the gene set, in which known and novel, coding and noncoding transcripts were included.

Gene Quantification Differential Expression Analysis: Expression level of gene was calculated by RSEM (v1.3.1) [3]. The heatmap was drawn by pheatmap (v1.0.8) according to the gene expression difference in different samples. Essentially, differential expression analysis was performed using the DESeq2(v1.4.5) [4] (or DEGseq[5] or PoissonDis)with Q value ≤ 0.05 (or FDR ≤ 0.001).

Gene Annotation: To take insight to the change of phenotype, GO (http://www.geneontology.org/) and KEGG (https://www.kegg.jp/) enrichment analysis of annotated different expression gene was performed by Phyper (https://en.wikipedia.org/wiki/Hypergeometric_distribution) based on Hypergeometric test. The significant levels of terms and pathways were corrected by Q value with a rigorous threshold (Q value ≤ 0.05).

**
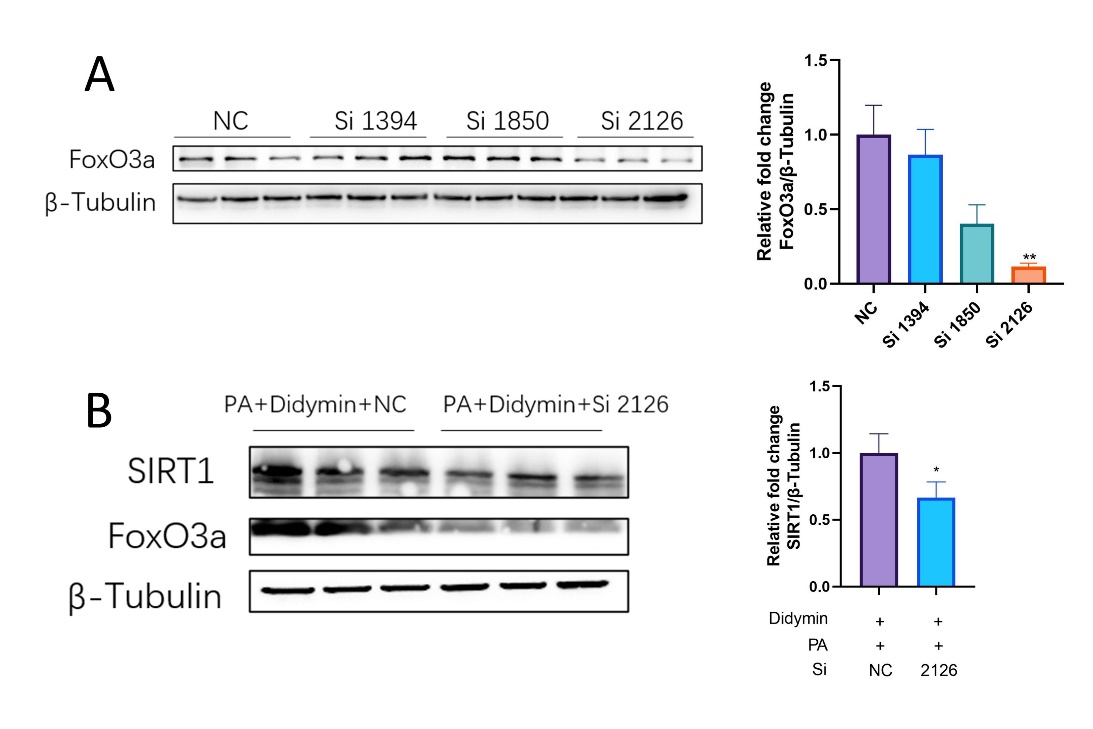
**

**Figure S1**. Didymin promotes the expression of Sirt1 by activating FoxO3a. (A) Western blot analysis of FoxO3a proteins in AML12 cells after siRNA transfection (n=3). (B) Western blot analysis of Sirt1 and FoxO3a proteins in AML12 cells after siRNA transfection (n=3). Data are expressed as mean ± SD. *P < 0.05, **P < 0.01


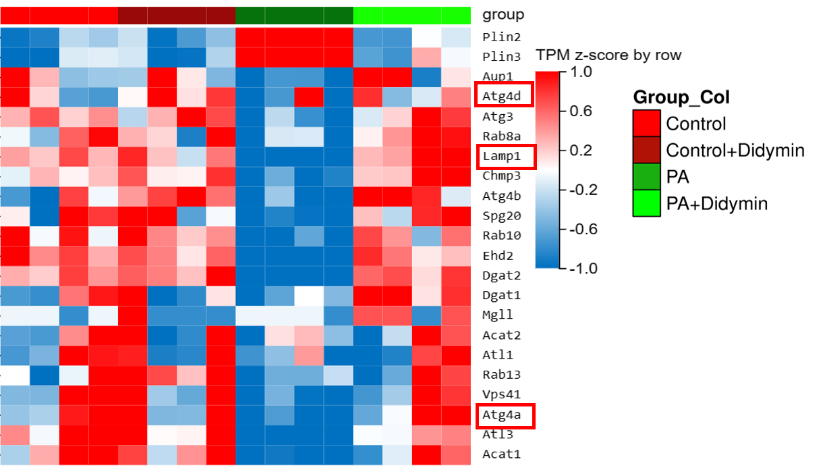


**Figure S2.** Heatmap of lipophagy-related genes. The red boxes indicate proteins directly regulated by FoxO3a, histone H4 K16, and TFEB.


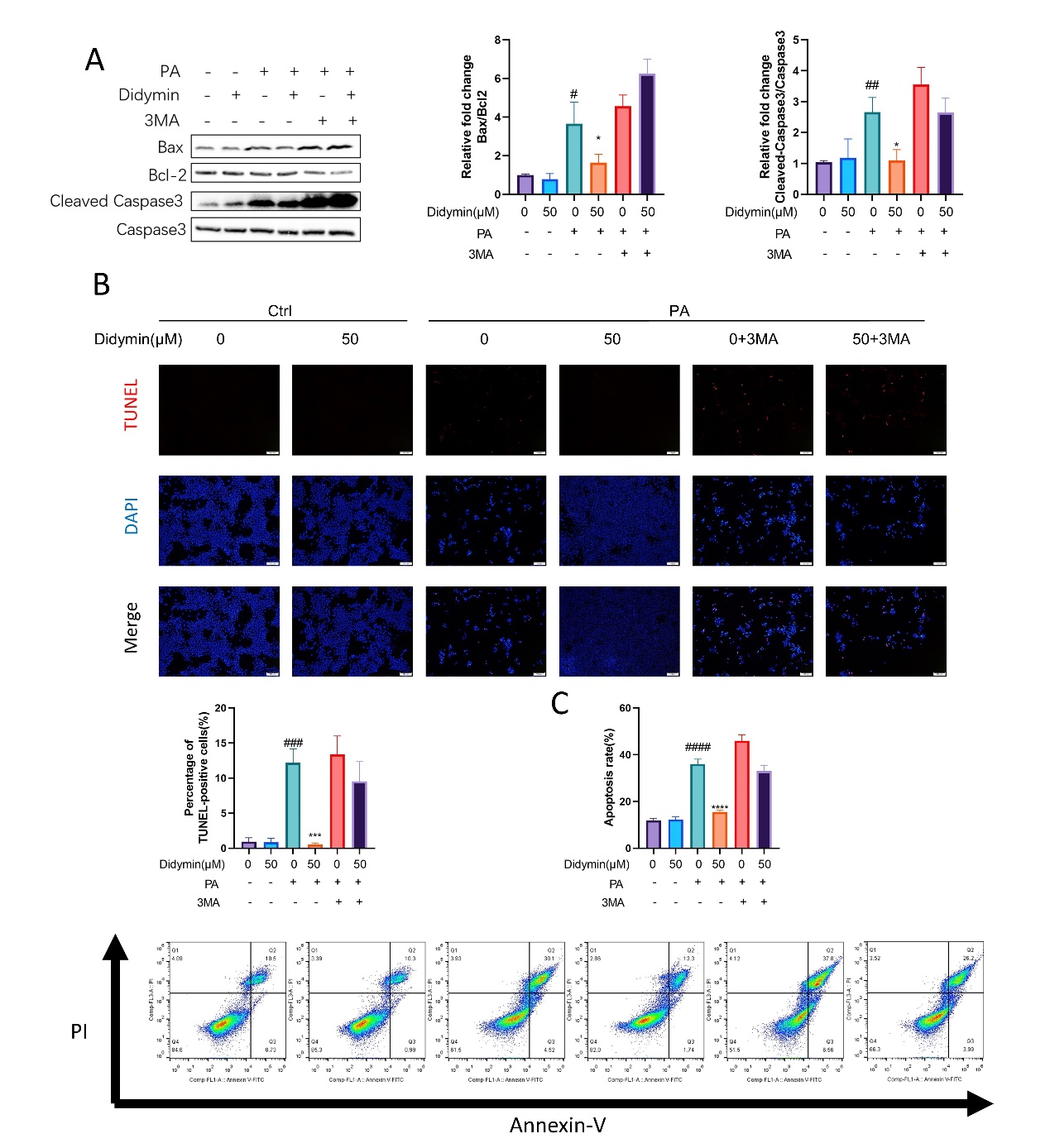


**Figure S3.** Didymin suppresses apoptosis by restoring lipophagy in PA-treated AML12 cells. (A) Representative images of TUNEL staining in AML12 cells (Scale bar = 100 μm). Quantification of the percentage of TUNEL-positive cells (n=3). (B) Apoptosis analysis of AML12 cells by flow cytometry. The results of quantitative analyses of apoptosis rate (n=3). (C) Western blot analysis of Bax, Bcl2, cleaved-caspase3, and caspase3 proteins in AML12 cells (n = 3). Data are expressed as mean ± SD. *P < 0.05, ***P < 0.001 PA vs. PA+Didymin. # P < 0.05, ## P < 0.01, ### P < 0.001, #### P < 0.0001 control vs. PA.


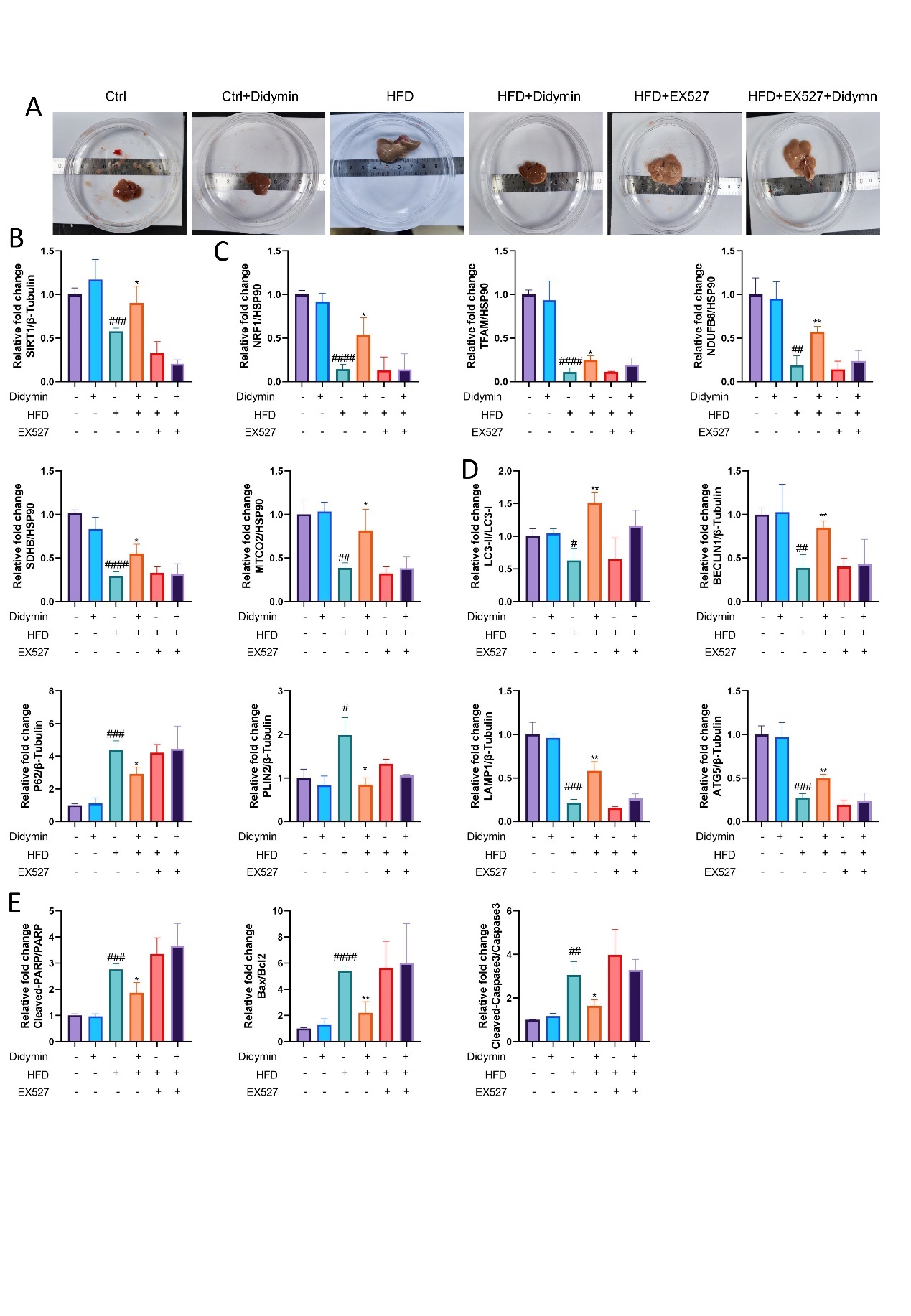


**Figure S4.** Liver morphology and statistical analysis of western blot results. (A) Liver morphology (B) Statistical analysis of western blot results in Figure 7A (n=3). (C) Statistical analysis of western blot results in Figure 7B (n=3). (D) Statistical analysis of western blot results in Figure 7E (n=3). (E) Statistical analysis of western blot results in Figure 7F (n=3). Data are expressed as mean ± SD. *P < 0.05, **P < 0.01 MAFLD vs. MAFLD+Didymin. # P < 0.05, ## P < 0.01, ### P < 0.001, #### P < 0.0001 control vs. MAFLD.

**
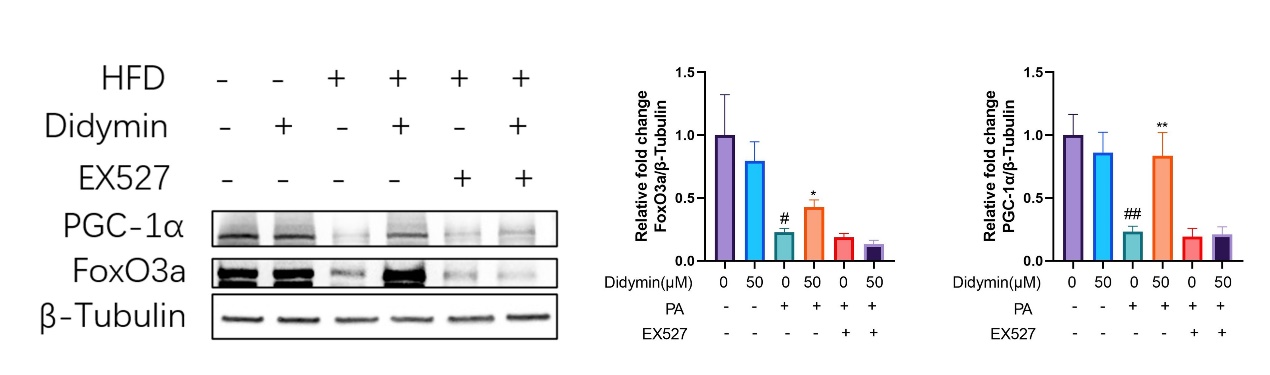
**

**Figure S5.** Western blot analysis of PGC-1α and FoxO3a in hepatocytes (n=3). Data are expressed as mean ± SD. *P < 0.05, **P < 0.01 MAFLD vs. MAFLD+Didymin. # P < 0.05, ## P < 0.01 control vs. MAFLD.


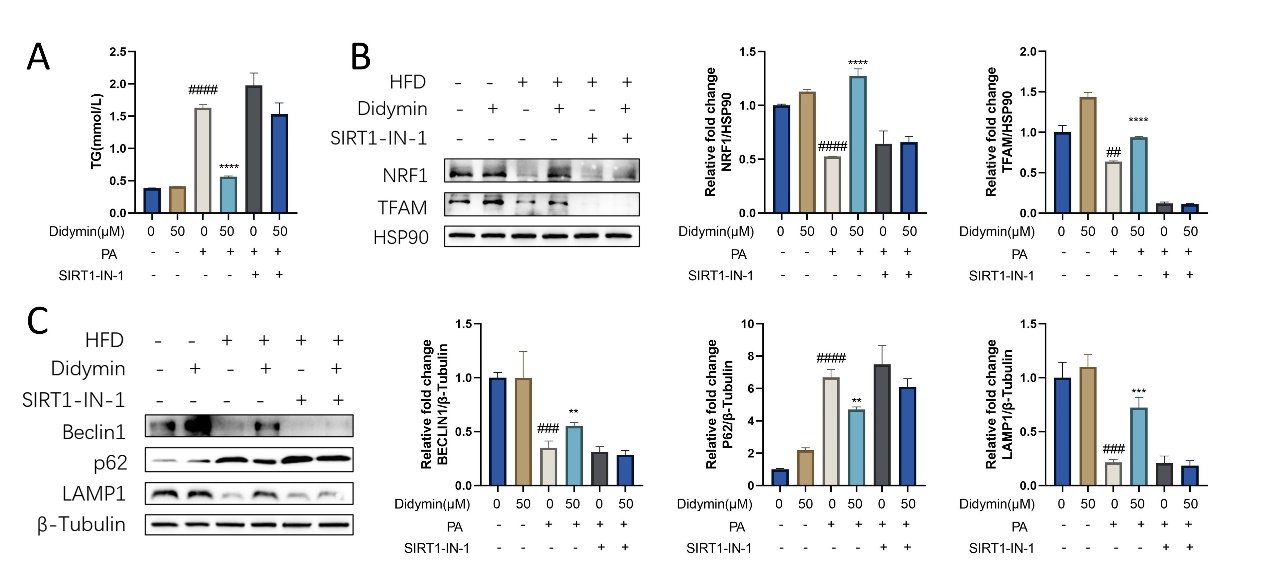


**Figure S6.** Sirt1-in-1 inhibits the activation of SIRT1 by Didymin in AML12 cells. (A) TG contents in AML12 cells (n=4). (B) Western blot analysis of NRF1 and TFAM in hepatocytes (n=3). (C) Western blot analysis of Beclin1, p62, and LAMP1 in hepatocytes (n=3). Data are expressed as mean ± SD. **P < 0.01, ***P < 0.001, ****P< 0.0001 PA vs. PA+Didymin. ## P < 0.01, ### P < 0.001, #### P < 0.0001 control vs. PA.

**
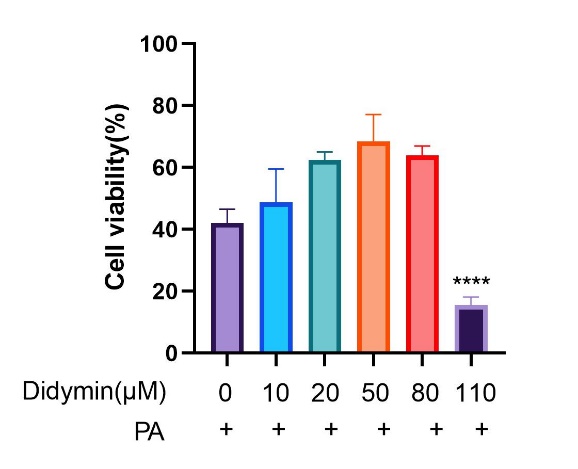
**

**Figure S7.** Cell viability of AML12 cells treated with PA and different concentrations of Didymin (n=4). Data are expressed as mean ± SD. ****P< 0.0001 PA vs. PA+Didymin.

1. Li, R., et al., *SOAP: short oligonucleotide alignment program.* Bioinformatics, 2008. **24**(5): p. 713-4.

2. Langmead, B. and S.L. Salzberg, *Fast gapped-read alignment with Bowtie 2.* Nat Methods, 2012. **9**(4): p. 357-9.

3. Li, B. and C.N. Dewey, *RSEM: accurate transcript quantification from RNA-Seq data with or without a reference genome.* BMC Bioinformatics, 2011. **12**: p. 323.

4. Love, M.I., W. Huber, and S. Anders, *Moderated estimation of fold change and dispersion for RNA-seq data with DESeq2.* Genome Biol, 2014. **15**(12): p. 550.

5. Wang, L., et al., *DEGseq: an R package for identifying differentially expressed genes from RNA-seq data.* Bioinformatics, 2010. **26**(1): p. 136-8.
